# The *Xylella fastidiosa*-Resistant Olive Cultivar “Leccino” Has Stable Endophytic Microbiota during the Olive Quick Decline Syndrome (OQDS)

**DOI:** 10.3390/pathogens9010035

**Published:** 2019-12-31

**Authors:** Marzia Vergine, Joana B. Meyer, Massimiliano Cardinale, Erika Sabella, Martin Hartmann, Paolo Cherubini, Luigi De Bellis, Andrea Luvisi

**Affiliations:** 1 Department of Biological and Environmental Sciences and Technologies, University of Salento, Via Prov. le Monteroni, I-73100 Lecce, Italy; marzia.vergine@unisalento.it (M.V.); erika.sabella@unisalento.it (E.S.); luigi.debellis@unisalento.it (L.D.B.); andrea.luvisi@unisalento.it (A.L.); 2 Federal Office for the Environment FOEN, Worblentalstrasse 68, CH-3063 Ittigen, Switzerland; joana.meyer@bafu.admin.ch; 3 Sustainable Agroecosystems, Institute of Agricultural Sciences, Department of Environmental Systems Science, ETH Zurich, Universitätsstrasse 2, CH-8092 Zurich, Switzerland; martin.hartmann@usys.ethz.ch; 4 WSL Swiss Federal Research Institute, Zürcherstrasse 111, CH-8903 Birmensdorf, Switzerland; paolo.cherubini@wsl.ch; 5 Department of Forest and Conservation Sciences, University of British Columbia, Vancouver, BC V6T 1Z4, Canada

**Keywords:** endophytes, *Olea europaea* microbiota, plant microbiome modulation, *Xylella fastidiosa*

## Abstract

*Xylella fastidiosa* is a highly virulent pathogen that causes Olive Quick Decline Syndrome (OQDS), which is currently devastating olive plantations in the Salento region (Apulia, Southern Italy). We explored the microbiome associated with *X. fastidiosa*-infected (*Xf*-infected) and -uninfected (*Xf*-uninfected) olive trees in Salento, to assess the level of dysbiosis and to get first insights into the potential role of microbial endophytes in protecting the host from the disease. The resistant cultivar “Leccino” was compared to the susceptible cultivar “Cellina di Nardò”, in order to identify microbial taxa and parameters potentially involved in resistance mechanisms. Metabarcoding of 16S rRNA genes and fungal ITS2 was used to characterize both total and endophytic microbiota in olive branches and leaves. “Cellina di Nardò” showed a drastic dysbiosis after *X. fastidiosa* infection, while “Leccino” (both infected and uninfected) maintained a similar microbiota. The genus *Pseudomonas* dominated all “Leccino” and *Xf*-uninfected “Cellina di Nardò” trees, whereas *Ammoniphilus* prevailed in *Xf-*infected “Cellina di Nardò”. Diversity of microbiota in *Xf*-uninfected “Leccino” was higher than in *Xf*-uninfected “Cellina di Nardò”. Several bacterial taxa specifically associated with “Leccino” showed potential interactions with *X. fastidiosa*. The maintenance of a healthy microbiota with higher diversity and the presence of cultivar-specific microbes might support the resistance of “Leccino” to *X. fastidiosa*. Such beneficial bacteria might be isolated in the future for biological treatment of the OQDS.

## 1. Introduction

The central role of the plant-associated microbiome in maintaining host’s fitness is being recognized more and more, and plants are now regarded as “holobionts”, which include the plant itself and the entire community of associated microbes, seen as a single unit of evolution [1,2]. The plant microbiome is known to be species- and cultivar-specific [3,4]; moreover, each plant habitat harbors its own specific microbiome [5]. Microorganisms interact with their host plants in several ways, from specific symbioses to relatively nonspecific beneficial effects, including plant growth promotion and protection from phytopathogens [6,7]. Like most plant species studied so far [8], olive (*Olea europaea* L.) has also an associated microbiome, which was shown to be cultivar-specific [9,10,11]. Being a tree species growing in arid and semiarid regions, olive might have established positive relationships with microbes to profit from several beneficial functions, including disease alleviation [12]. Indeed, microbes isolated from olive plants showed ability to inhibit phytopathogens, at least in in vitro assays [13]. In contrast with animals and humans, very little is known about the role of microbiome in plants and their response to plant diseases. Passos da Silva et al. [14] analyzed ten olive knots infected by *Pseudomonas savastanoi* pv. *savastanoi* and showed the presence of a highly diverse bacterial microbiota. However, they did not analyze *P. savastanoi*-uninfected plants as control, which makes it impossible to derive conclusions about the response of the native olive microbiome to the disease.

One of the most dramatic diseases for olive trees is the Olive Quick Decline Syndrome (OQDS), which resulted in devastation of thousands of hectares of plants in the Salento region, in Southern Italy), including very old trees [15,16], and infected more than twenty other plant species in the region [17]. Future projections suggested that the pathogen will persist in Europe [18]. It is caused by a strain (named “De Donno”, previously known as “CoDiRO”) of *Xylella fastidiosa* Wells et al., subsp. *pauca*, a xylem-limited bacterial pathogen transmitted in olive trees by sap-feeding insect vectors, i.e., the meadow spittlebug (*Philaenus spumarius* L.) [18]. *X. fastidiosa* can infect more than 550 plant species, including trees of major importance in forest ecosystems and urban greening plantations, such as oak, elm, sycamore, and maple, representing one of the major threats to agro-forest-ecosystems all over the world [19,20,21,22]. The pathogen invades the xylem, resulting in the occlusion of the vessels with subsequent restriction of water movement, inducing the related parts of tree crown to dry out [23,24,25,26]. To date, the most widespread olive cultivars in the Salento region, “Cellina di Nardò” and Ogliarola di Lecce, showed high sensitivity to *X. fastidiosa*, while a notable resistance was observed in the less common cultivar “Leccino” [27,28,29].

The mechanisms of resistance of the “Leccino” trees are still unclear. It was previously shown that “Leccino” resistance is probably influenced by the lignin amount in the xylem vessels, which can limit the bacteria movement and the host invasion by slowing down the disease progression [30], or by the constitutive amount of secondary metabolites such as hydroxytyrosol glucoside [29]. However, in a citrus plant or grapevine affected by *X. fastidiosa*, it was shown that the nature of the endophytic bacterial community is able to downregulate the pathogen growth or plant symptoms, either because they compete with the pathogen or because they secrete substances able to modulate its virulence [31,32]. The first results on olive tree microbiome were recently released, and they indicated that a very high proportion of the detected fungi occurs in the resistant cultivar FS17 [33].

Microbial diversity is associated with plant health and productivity [34,35], similarly to the gut microbiome in humans [36]. It is possible that the autochthonous microbiome associated with “Leccino” trees contributes to its resistance to *X. fastidiosa*, for example, by inhibiting the pathogen via microbe–microbe interactions or by triggering the reaction of the plant immune system. This interaction could synergistically enforce the natural resistance of “Leccino” trees to this pathogen.

In this study, we explored and compared the autochthonous fungal and bacterial microbiota associated with both *X. fastidiosa*-infected (*Xf*-infected) and -uninfected (*Xf*-uninfected) “Leccino” trees (*X. fastidiosa*-resistant), using the *X. fastidiosa*-susceptible “Cellina di Nardò” trees as a control. Our aims were as follows: (i) to assess differences in terms of assemblage, diversity, and structure between the two cultivars; (ii) to understand the response of the native microbiota to the *X. fastidiosa* infection; and (iii) to identify taxa specifically associated to the cultivar “Leccino”. Moreover, we explored the microbial co-occurrence/co-exclusion network within the endophytes of *Xf*-infected “Leccino”, to gain insights into the multispecific interactions involving *X. fastidiosa*. We hypothesized the following: (i) the cultivar “Leccino” harbors specific microbiota, with a different assemblage and a higher diversity compared to “Cellina di Nardò”; (ii) the response of the olive microbiota to *X. fastidiosa* infection is different between the two cultivars; (iii) microbial taxa exist that are maintained, or that appear only, during *X. fastidiosa* infection in “Leccino” but not in “Cellina di Nardò”; and (iv) these “Leccino”-specific taxa show potential multispecific interactions with *X. fastidiosa*.

## 2. Results

### 2.1. 16S rRNA Gene and ITS2 Illumina Sequencing Output

A total of 4,548,524 raw sequences were obtained from the 16S rRNA gene amplicon libraries after joining the paired ends, from 78 of the 80 samples (two samples did not give a sequencing output). After length and quality filtering, a total of 4,534,886 sequences remained, of which 4,337,856 (95.7%) were identified as non-chimeric. They were grouped into 21,358 OTUs at 97% sequence similarity threshold. After removal of mitochondrial, plastidic, unassigned OTUs, and OTUs with less than 10 reads, 881 OTUs remained, representing a total of 3,944,950 sequences (2806–121,289 sequences per sample, with an average of 50,576.3 ± 28,172.1; Table S1). 

The ITS2 amplicon libraries produced a total of 3,536,039 raw sequences from 76 of the 80 samples (four samples did not give a sequencing output), after joining the paired ends. After length and quality filtering, a total of 2,868,275 sequences remained, of which 2,753,359 were identified as non-chimeric and 2,753,353 (96.0%) were extracted as ITS2. They were grouped into 6039 OTUs at 97% sequence similarity threshold. After removal of unassigned OTUs, metazoan OTUs and OTUs with less than 10 reads, 1006 OTUs remained, representing a total of 2,620,425 sequences (384–104,659 sequences per sample, with an average of 34,479.3 ± 28,405.5; Table S1). 

Before proceeding with the sequence analysis, five 16S rRNA gene samples and nine ITS2 samples were eliminated because of the lower number of reads in order to increase the sequencing depth at the rarefaction step (11950 reads for Bacteria and 4850 for Fungi), thus optimizing the per-sample coverage. However, the statistical design was nor unbalanced, since for each type of sample (*Cultivar* × *Habitat* × *Microbiota* × *Infection status*) three to five biological replicates remained (Table S2), thus allowing a full factorial statistical analysis for both Prokaryotes and Fungi. After removal of these samples, the number of OTUs was reduced to 875 for Bacteria and 974 for Fungi. The sequences were submitted to EMBL (www.ebi.ac.uk/ena) under the project number PRJEB32050.

### 2.2. Sequence Analysis

For all samples, Good’s coverage was >99.5% for Prokaryotes (average = 99.84%) and >98.7% for Fungi (average = 99.57%) (Figure S1), demonstrating that most of the samples’ microbial diversity was captured.

**Figure 1:**
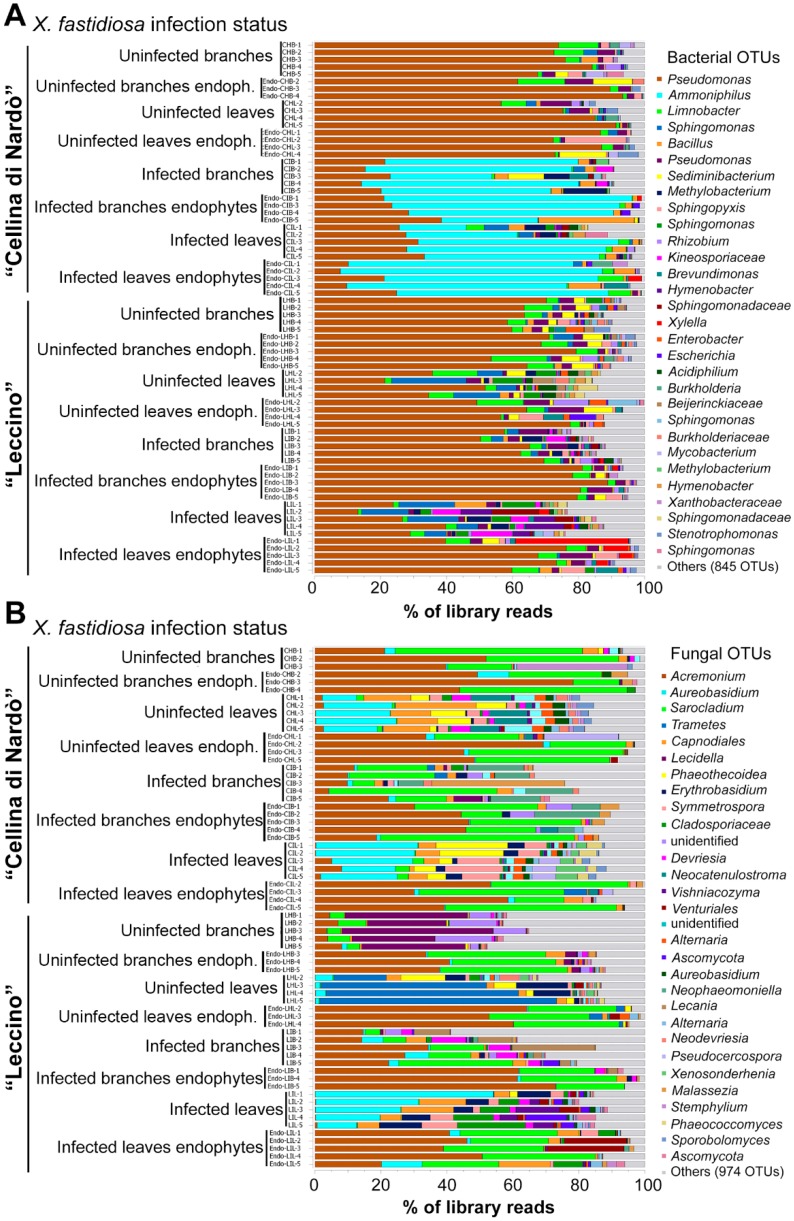
Taxonomy map of the olive microbiome. The graph shows the 30 most abundant bacterial (**A**) and fungal (**B**) OTUs, named according to the best achieved identification. Samples are grouped according to the olive cultivar and then to the sample type.

The taxonomic assignment of the prokaryotic OTUs revealed 14 phyla, 25 classes, 66 orders, 98 families, and 184 genera (Table S3), all of them bacterial (Archaea were not detected). The most dominant phylum was *Proteobacteria* with an average relative abundance per sample of 76.0%, followed by *Firmicutes* (16.9%), *Bacteroidetes* (3.8%), and *Actinobacteria* (2.8%). The remaining phyla (*Planctomycetes*, *Chloroflexi*, *Verrucomicrobia*, *Acidobacteria*, *Patescibacteria*, *Cyanobacteria*, *Chlamydiae*, *Armatimonadetes*, Candidate division *FBP,* and *Nitrospirae*) accounted for a total of 0.49%. The most abundant families were *Pseudomonadaceae* (56.2%) and *Paenibacillaceae* (14.8%) (Table S3). The ten most abundant OTUs accounted for 83.9% of all sequences, and the 30 most abundant ones accounted for 92.4% (Figure 1A). All samples were dominated by *Pseudomonas*, related to the species *P. aeruginosa*, (98.7%), *P. stutzeri* (98.9%) and, at a less extent, *P. taiwanensis/monteilii/knackmussii/juntendi* (98.7%), except the *Xf*-infected “Cellina di Nardò” samples that were dominated by *Ammoniphilus* (Figure 1A). *X. fastidiosa* was the 10th most abundant taxon. It was detected in all “Leccino” *Xf*-infected samples (Figure S2), where it was present in 16 out of 20 individual samples (Figure 1A; Table S3), and in the *Xf*-infected “Cellina di Nardò” samples only within the endophytic community (Figure S2), where it appeared rather erratic among the individual samples (Figure 1A).

The taxonomic assignment of the fungal OTUs revealed 5 phyla (one of which remained unidentified), 26 classes, 63 orders, 142 families, and 241 genera (Table S4). The most dominant phylum was *Ascomycota,* with an average relative abundance per sample of 84.4%, followed by *Basidiomycota* (11.5%) and unidentified (4.1%). The most abundant families were *Hypocreales_fam_Incertae_sedis* (45.8%) and *Aureobasidiaceae* (7.3%) (Table S4). The ten most abundant OTUs accounted for 64.7% of all sequences, and the 30 most abundant ones accounted for 81.8% (Figure 1B). All endophytic communities of both cultivars appeared very similar, with dominant *Acremonium* and *Sarocladium* (Figure 1B). Total community (epiphytes + endophytes) appeared very similar between *Xf*-infected and -uninfected “Cellina di Nardò” samples, with marked differences between habitats (leaves and branches); branches looked similar to the endophytic communities, while, in leaves, *Aureobasidium* and *Capnodiales* prevail (Figure 1B). “Leccino” showed a clearly different fungal community in both *Xf*-uninfected leaves and branches (with dominant *Trametes* and *Lecidella*, respectively) compared to “Cellina di Nardò”. However, the *Xf*-infected “Leccino” looked very similar to the *Xf*-infected “Cellina di Nardò” (Figure 1B).

**Figure 2:**
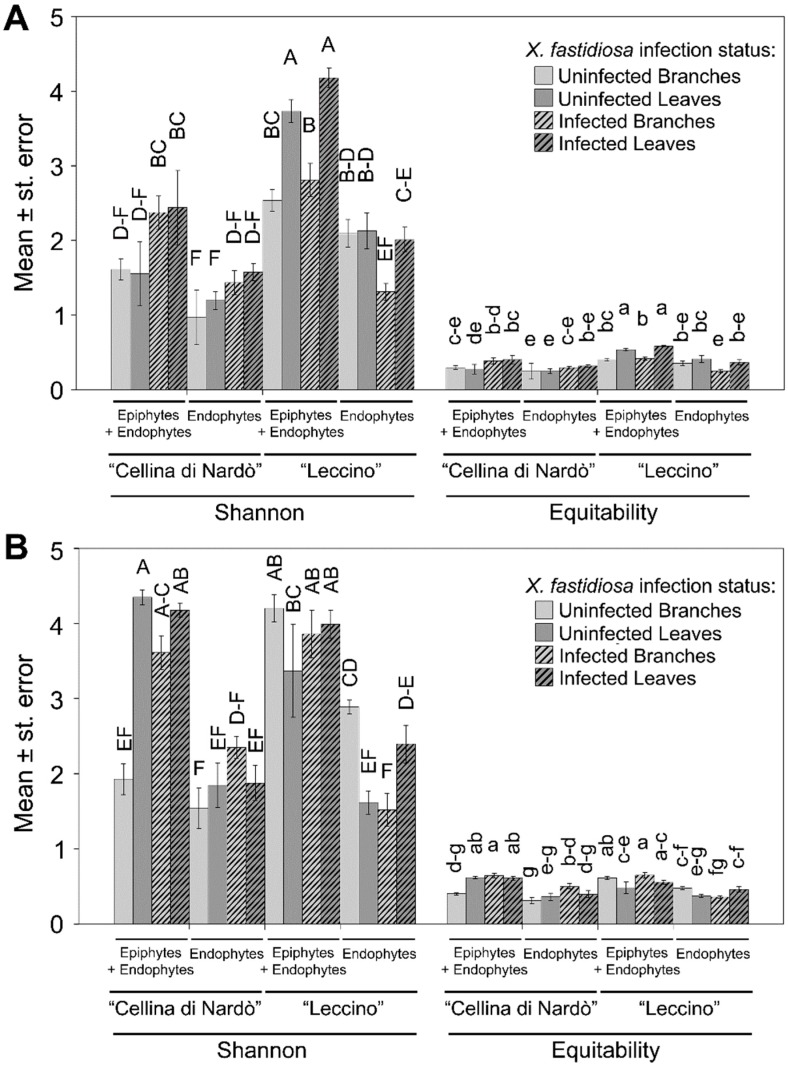
Alpha-diversity metrics. Shannon–Wiener and Equitability indices for bacterial (**A**) and fungal (**B**) microbiota in olive, grouped per sample type and ordered by cultivar. Different letters indicate significantly different means (factorial ANOVA followed by Duncan’s post hoc test, *p* < 0.05).

Alpha-diversity metrics (Shannon diversity and equitability) of the endophytes were lower when compared to the total community for both fungi and bacteria, as expected (*p* < 0.001; Figure 2). “Leccino” showed a higher bacterial diversity than “Cellina di Nardò” (*p <* 0.001) (Figure 2A), while, for fungi, this difference was strongly reduced (*p* = 0.044) (Figure 2B). Habitat and infection status also affected the bacterial diversity significantly, with higher indices found in leaves (*p* < 0.001) and in *Xf*-infected samples (*p* = 0.018), respectively. Both factors did not significantly affect fungal diversity instead (*p* = 0.11 and 0.056, respectively). Significant interactions for bacteria were Cultivar*Infection status (*p* = 0.007) and Cultivar*Habitat (*p* = 0.003). For fungi, also, the same interactions were significant (*p* = 0.014 and *p* < 0.001, respectively), as well as Cultivar*Infection status*Habitat (*p* < 0.001). Interestingly, *Xf*-uninfected “Leccino” showed a notably higher bacterial diversity compared to the corresponding *Xf*-uninfected “Cellina di Nardò”, for both total community and endophytes (Figure 2A). In the case of fungi, this difference appeared only in branches but not in leaves (Figure 2B). The Equitability index followed the same trend as the Shannon index.

**Figure 3:**
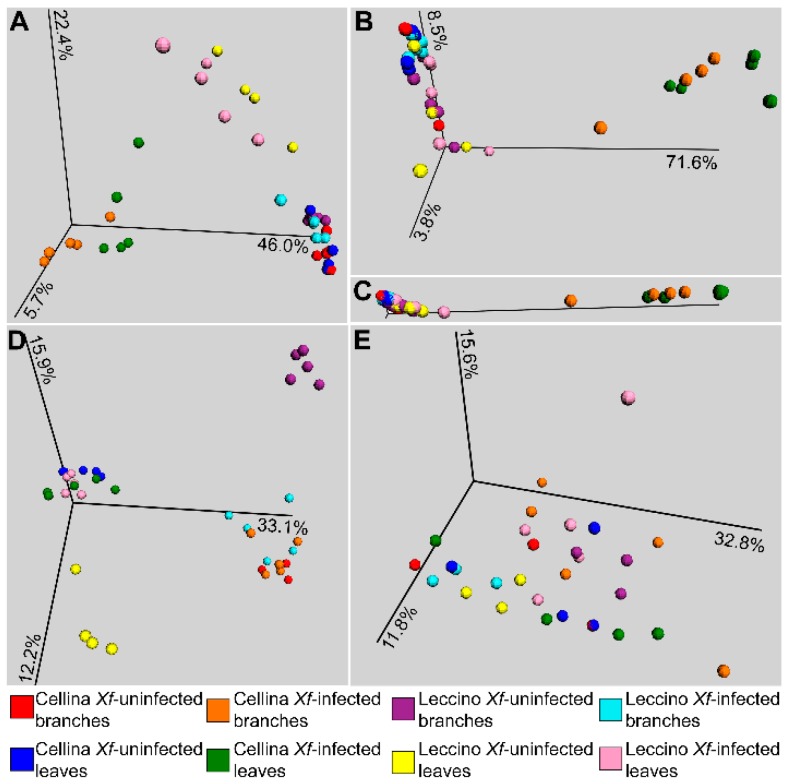
Beta-diversity analysis of the microbiota associated to the olive cultivars “Leccino” and “Cellina di Nardò”. Principal Component Analysis (PCoA) plots of the Bray–Curtis distances between bacterial epiphytic + endophytic microbiota (**A**), bacterial endophytic microbiota (**B**), bacterial endophytic microbiota, with plot scaled according to the % of variance explained by the axes (**C**), fungal epiphytic + endophytic microbiota (**D**), and fungal endophytic microbiota (**E**). *Xf* = *Xylella fastidiosa*.

Beta-diversity analysis was performed with Bray–Curtis dissimilarities. The global score-heatmap clearly showed that the bacterial microbiota of *Xf*-infected “Cellina di Nardò” plants was the most different from all the other ones, especially the leaf endophytic microbiota (Figures S3 and S4A). In contrast, the *Xf*-infected “Leccino” samples maintained a higher similarity with the *Xf*-uninfected samples in the case of the endophytic microbiota (Figures S3 and S4A). This trend was not observed for the fungal microbiota (Figures S5 and S4B). Total microbiota (epiphytes + endophytes) and endophytes were then separately plotted (PCoA plots, Figure 3) and analyzed for significance of factors. The structure of the total bacterial microbiota was significantly different between *Xf*-infected and -uninfected plants (Adonis test, *p* < 0.001) (Figure 3A; Table 1). However, the separate analysis of each cultivar showed that only the “Cellina di Nardò” microbiota was strongly affected by the infection status, while *Xf*-infected “Leccino” samples did not differ significantly from the *Xf*-uninfected “Leccino” (*p* = 0.067; Table 1). The same trend was observed for the endophytes, where the difference of the two cultivars was even clearer: all samples clustered together except the *Xf*-infected “Cellina di Nardò” (Figure 3B). The effect size of the factor “infection status” was very large here, as it can be appreciated by drawing the plot according to a proportionated axes scale (Figure 3C). Separate testing of branches and leaves showed that the endophytic microbiota of the leaves of “Leccino” had a particular stability to the *X. fastidiosa* infection (Adonis *p* = 0.429 for the factor “infection status” of “Leccino” leaves’ endophytes; Table 1). In general, the bacterial microbiota was significantly different between “Leccino” and “Cellina di Nardò” cultivars (Table 1). However, this difference was much more pronounced for *Xf*-infected (*p* < 0.001) than for *Xf*-uninfected plants (*p* = 0.04). Indeed, analyzing separately *Xf*-uninfected branches and leaves, no difference was found between endophytes of “Cellina di Nardò” and “Leccino” (*p* > 0.1 for the factor “cultivar”; Table 1). The total fungal microbiota showed an opposite behavior (which was indeed evident already after identification of the dominant OTUs, Figure 1B): samples clustered according to habitat but *Xf*-uninfected “Leccino” branches and leaves were clearly different from other samples (Figure 3D).

However, *Xf*-infected “Leccino” branches and leaves showed the same structure than “Cellina di Nardò” plants (Figure 3D). Therefore, only a low significant effect of the infection status was found between all samples (*p* = 0.024), which was due only to “Leccino” (*p* < 0.001), while “Cellina di Nardò” *Xf*-infected and -uninfected samples did not differ (*p* = 0.069) (Table 1). In the case of fungal endophytes (Figure 3E), no clear pattern could be identified, as confirmed by the generally not significant Adonis *p*-values (Table 1).

### 2.3. Identification of Significant Taxa

**Figure 4:**
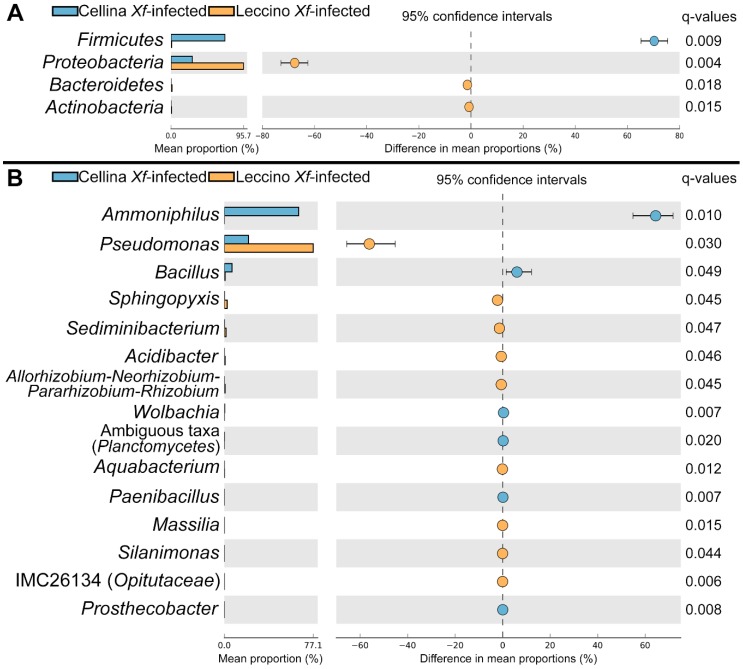
Extended error plots showing phyla (**A**) and genera (**B**) of bacterial endophytes significantly different between *Xf*-infected “Cellina di Nardò” and “Leccino” olive cultivars, according to White’s nonparametric test. FDR-corrected *p*-values (q-values) are shown. Taxa are ordered according to the effect size of the difference. *Xf* = *Xylella fastidiosa*.

Considering the results of the beta-diversity analysis, which indicated that the bacterial microbiota is similar between *Xf*-infected and -uninfected “Leccino” trees, while it is significantly different between *Xf*-infected and -uninfected “Cellina di Nardò” trees (Table 1 and Figure 3B,C), the identification of significant taxa was focused on bacteria only. Between *Xf*-infected “Leccino” and “Cellina di Nardò” endophytic bacterial microbiota, four phyla were significantly different (White’s test, FDR-corrected *p* < 0.05): *Firmicutes* were more abundant in “Cellina di Nardò” *Xf*-infected samples, while *Proteobacteria*, *Bacteroidetes* and *Actinobacteria* were more abundant in “Leccino” (Figure 4A). Six genera were enriched in *Xf*-infected “Cellina di Nardò”, and the dominant of which was by far *Ammoniphilus* (Figure 4B). Nine genera were enriched in “Leccino” samples instead, the most abundant of which was *Pseudomonas* (Figure 4B).

**Figure 5:**
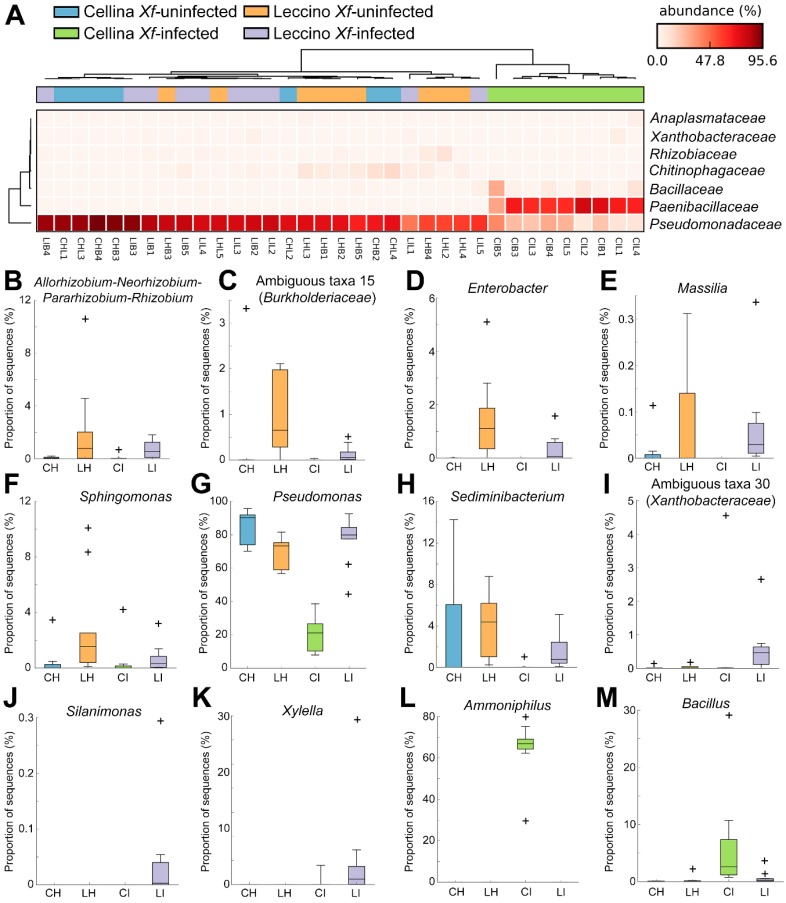
Bacterial families (**A**) and genera (**B**–**M**) significantly different between *Xf*-infected and -uninfected, “Cellina di Nardò” and “Leccino” olive cultivars (endophytes only), according to Kruskal–Wallis test at FDR-corrected *p* < 0.05. *Xf* = *Xylella fastidiosa*. CH = *Xf*-uninfected “Cellina di Nardò”; LH = *Xf*-uninfected “Leccino”; CI = *Xf*-infected “Cellina di Nardò”; LI = *Xf*-infected “Leccino”; “+” signs indicate outliers.

To identify taxa potentially involved in the resistance of “Leccino” to *X. fastidiosa*, we compared *Xf*-infected and -uninfected, “Leccino” and “Cellina di Nardò” cultivars (endophytes only). Seven bacterial families showed a significantly different distribution among the groups (Kruskal–Wallis test, FDR-corrected *p* < 0.05); their clustering showed how “Cellina di Nardò” *Xf*-infected samples clustered apart (Figure 5A). Seventeen genera were significantly different; in particular, *Allorhizobium–Rhizobium–Pararhizobium–Rhizobium*, *Massilia*, *Enterobacter*, *Sphingomonas,* and an unidentified *Burkholderiaceae* genus were found only in *Xf*-infected and -uninfected “Leccino” (Figure 5B–F).

*Pseudomonas* and *Sediminibacterium* were present also in *Xf*-uninfected “Cellina di Nardò” but significantly reduced or disappeared after *X. fastidiosa* infection. In “Leccino”, instead, they persisted also in *Xf*-infected plants (Figure 5G,H). *Silanimonas* and an unidentified *Xanthomonadaceae* genus were detected only in *Xf*-infected “Leccino” (Figure 5I,J). *X. fastidiosa* was also significantly different between groups, being more abundant in *Xf*-infected “Leccino” (Figure 5K; Figure S2). *Ammoniphilus* and *Bacillus* characterized the “Cellina di Nardò” *Xf*-infected samples (Figure 5L,M). 

### 2.4. Correlation of Co–Occurrence Patterns and Network Analysis

To identify taxa potentially interacting with *X. fastidiosa* within the leaf endophytic microbiota of *Xf*-infected “Leccino” leaves, we created a network based on correlation of co-occurrence patterns [37].

**Figure 6:**
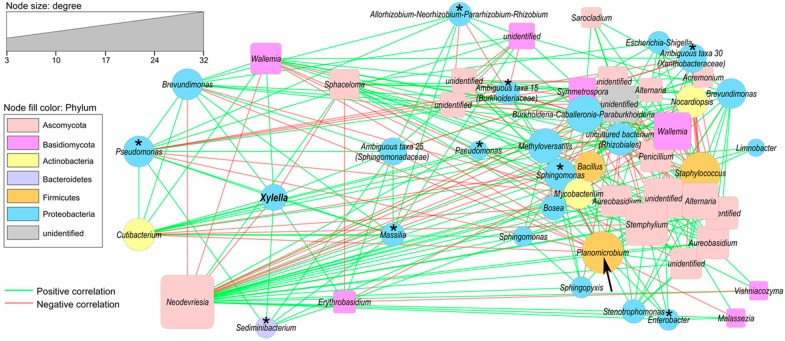
Co-occurrence network of the endophytic microbiota of “Leccino” leaves infected with *Xylella fastidiosa*. Node shape represents kingdom (circle = bacterial OTUs, squares = fungal OTUs), while size represents the degree (number of connections) according to the legend. Nodes were colored by phylum and labeled according to the lowest identified taxonomic level. Edges (connections) are colored green (positive) or red (negative), and they represent highly significant correlations according to four similarity measures (see Section 4 for details). Asterisks indicate taxa significantly enriched in “Leccino” (see Figure 5B–J). Arrow indicates the hub OTU *Planomicrobium*. The network layout was generated with the edge-forced spring-embedded algorithm, except for the *X. fastidiosa* cluster (*Xylella* OTU + first degree nodes) that was drawn separately with a circular layout for easy of interpretation (the original network is provided in Figure S6).

Out of 43 bacterial and 47 fungal OTUs that passed the minimum abundance thresholds, 28 and 24 showed at least one potential interaction. There were 225 positive and 192 negative interactions that were supported by at least three of the four correlation and distance methods computed (Figure 6). There was no significant difference in the average degree per OTU (number of connections) between fungi and bacteria (*t*-test, *p* = 0.30). *X. fastidiosa* was directly interacting with 10 OTUs (six bacterial and four fungal), showing eight positive and two negative interactions (in order to facilitate interpretation of the network, we arranged the *X. fastidiosa*-cluster separately with a circular layout; however, the original network can be seen in Figure S6). The fungal OTUs identified as *Sphaceloma* and *Neodevriesia* showed co-exclusion with *X. fastidiosa*; however, they were not significantly different between cultivars (FDR-corrected *p* > 0.1). Interestingly, several OTUs belonging to the bacterial genera that were found to be significantly associated with “Leccino” (Figure 5B–J), were found interacting (directly or indirectly) with *X. fastidiosa* (Figure 6, asterisks). “Hub” OTUs, i.e., important taxa identified according to degree and betweenness centrality values were the fungal OTU *Neodevriesia* and the bacterial OTU *Planomicrobium* (Figure 6, arrow); the latter showed an association trend to “Leccino” similar to the other “Leccino”-specific taxa (Figure S7), although not significant (FDR-corrected *p* = 0.12); however, g-test of independence indicated that, also, this OTUs was non-randomly distributed between *Xf*-infected cultivars (FDR-corrected *p* < 0.001).

## 3. Discussion 

In this work, we aimed to characterize the microbiota associated with the *X. fastidiosa*-resistant olive cultivar “Leccino”, a highly promising cultivar able to survive and grow in Salento despite the severe *X. fastidiosa* outbreak that affects this region of Italy since several years [38]. So far, no effective method was found to control the pathogen [22,39,40], but only several conventional and innovative diagnostic approaches were tested for the De Donno strain [28,41,42]. It is of primary importance to understand the basic resistance mechanisms of “Leccino”, in order to implement successful agronomical strategies to overcome the current critical situation of the disease. The working hypothesis of this study was that the autochthonous microbiota associated with the cultivar “Leccino” plays a role in the cultivar’s resistance to *X. fastidiosa*, perhaps acting synergistically with the plant’s own resistance mechanisms, as suggested for the olive cultivar FS17 [33]. Plant microbiomes are species- and cultivar-specific, and therefore the final desired goal of our research is to identify microbial species or consortia, specifically associated to “Leccino” that could be used for the biological control of the Olive Quick Decline Syndrome. First, we characterized the microbiome associated with branches and leaves of the cultivar “Leccino”. To do this, we applied a cultivation-independent approach (Illumina sequencing) and used an *X. fastidiosa*-susceptible cultivar (“Cellina di Nardò”) as control for comparison. Our sampling strategy followed a full factorial scheme, including the separation of endo + epiphytic microbiota and endophytic only (by surface-sterilizing a subset of samples), the differentiation between branches and leaves, and the analysis of *Xf*-infected and -uninfected plants. Both Prokaryotic and Fungal microbiota were analyzed. To the best of our knowledge, no data have been available so far in the literature on the total microbiome associated with *Xf*-infected and *Xf*-uninfected olive trees in Salento. 

The *Xf*-infected and -uninfected samples of our study were collected from two areas within the same natural park, at 3 km distance from each other. This was a strategical choice, due to the higher risk to select false-negative samples in orchards in which positive plants were previously detected compared to sampling in orchards in which the pathogen was not yet ascertained. This event is not uncommon for an erratically distributed pathogen with a long period of latency [22]. However, the sampling area is flat, without surrounding or dividing orographic elements, which might influence notably the average weather conditions (Table S5). Although some micro-climatic conditions of the sampling sites (difficult to track) might have influenced the olive-associated microbiome, soil analysis showed largely similar pedological parameters (Table S6). Thus, pedo-climatic conditions can be reliably considered as homogeneous within the whole sampling area.

Our samples showed an erratic distribution of *X. fastidiosa*, especially in the branches, confirming previous reports [25,26,41]. The taxonomic composition of the microbiota showed hundreds of families and genera, which is in agreement with the data available from other tree species [43,44]. Dominant organisms were typical plant-associated taxa, also known to be endophytic, such as *Pseudomonas*, *Sphingomonas,* and *Methylobacterium* among bacteria, and *Acremonium*, *Aureobasidium,* and *Sarocladium*, among fungi. Interestingly, Archaea were not detected, although the primers used in this work are able to amplify archaeal 16S rRNA genes [44]. Müller et al. [9] analyzed the leaf microbiota of 10 *O. europaea* cultivars from the Mediterranean basin, including “Leccino”, and found abundant Archaea in all of them. However, these plants were all grown at a single agricultural site in Spain, and therefore this abundance might have been determined by the local soil and environmental conditions.

In our study, *Xf*-infected “Cellina di Nardò” samples appeared severely dysbiotic, especially for the leaf endophytic bacteria. Here the endophytic bacteria were dominated by *Ammoniphilus*, an obligate oxalotrophic bacterium that requires high concentration of ammonium to grow [45]. To the best of our knowledge, its presence in diseased plants has never been shown until now. Likely, its abundance in the *Xf*-infected “Cellina di Nardò” is linked to the advanced status of the disease in our samples, where high amount of ammonium might have accumulated due to tissue decay. The nitrogen level in the soil where *Xf*-infected plants grew is good (1.92 g kg^−1^); however, it is unlikely that a high concentration of ammonium in plant tissues could have derived directly from soil nitrogen, otherwise we would have observed a prevalence of *Ammoniphilus* also in the *Xf-*infected “Leccino” samples. *Xf*-infected “Leccino”, instead, showed a bacterial assemblage and structure similar to that of the *Xf*-uninfected samples, and this was particularly evident for the leaf endosphere. Considering that this is the primary site of *X. fastidiosa* infection, the stability of the endophytic microbiota in the leaves of *Xf*-infected “Leccino” appears very relevant and promising for the biological control of *X. fastidiosa*. Dysbiosis is the unbalanced microbial status associated to several diseases in humans and animals [46,47]. However, little is known about the equivalent situation in plants, and this is one of the first reports showing dysbiosis associated to a plant disease. We argue that the stability of the endophytic microbiota of “Leccino” during *X. fastidiosa* infection contributes to the maintenance of a good healthy status of the plant. In fact, it is known that the plant microbiome provides several ecological services important for the maintenance of the host’s fitness and health [48], within the concept of the “plant holobiont” [2,49], as it was shown, for example, in the case of the tomato var. Hawaii 7996’s resistance to *Ralstonia solanacearum* [50]. Moreover, the endophytes are expected to establish a more intimate relationship with the host than the epiphytes [8,51]. As such, the bacterial species forming the leaf endophytic microbiota of “Leccino” might be a promising source of strains with potential biocontrol activity against *X. fastidiosa*.

The microbiota of “Leccino” also showed a higher diversity and equitability with respect to those of “Cellina di Nardò”. Again, this difference was especially evident for bacteria of both, endo + epiphytic and endophytic microbiota (Figure 2). Microbial diversity was linked to the health and fitness of ecosystems in general [52,53] and plants in particular [54,55]. A well-balanced microbiome supports a series of functions that turns beneficial to the host, including the production of inhibitory compounds and growth-promoting factors, and can provide a “barrier effect” that limits both space and nutrients for potential alien species’ and pathogens’ growth [6]. We suggest that the more diverse “Leccino” microbiota reduces the effects of *X. fastidiosa* infection or modifies its output effect for the plant.

The total fungal microbiota appeared very diverse, but no clear difference could be seen in the *Xf*-infected samples (Figure 3D). The endophytic fungal community did not show clear distinguishable pattern between the two cultivars, and therefore we concentrated our analysis on the bacterial microbiota. A statistical comparison between the microbiota of the two cultivars indicated that some bacterial species occurred only in “Leccino”, either in both, *Xf*-infected and -uninfected plants, or only in the *Xf*-infected ones. These “Leccino”-specific taxa include species known to exert beneficial effects on the host plants, such as *Rhizobium* [56], *Burkholderiaceae* [57], *Sphingomonas* [58], and *Enterobacter* [59]. *Massilia* is a genus recently shown to be involved in plant-microbe interactions at root and rhizosphere level [60,61]; however, it was never shown so far in the plant endosphere or phyllosphere. *Pseudomonas* and *Sediminibacterium* were reduced in *Xf*-infected “Cellina di Nardò”, while the former is a very well-known plant beneficial genus [62], the latter is a typical sediment bacterium that was never shown associated to plants or beneficial. Although the genus *Pseudomonas* includes some phytopathogenic species (such as the mentioned *P. savastanoi* pv. *savastanoi*), in our study the detected *Pseudomonas* OTUs were taxonomically related mainly to different species, such as *P. aeruginosa*, *P. stutzeri* and, at a less extent, other *Pseudomonas* spp. (BLAST analysis of representative sequences). We hypothesize that these “Leccino”-specific taxa could interact with *X. fastidiosa* in “Leccino”, and therefore the next step was to perform a co-occurrence analysis to assess potential interactions in the endophytic microbial network.

Microbe–microbe interactions can change the net effect of a microbiome on the host, including plants [63] and animals [64]. Therefore, it is of primary importance to investigate microbial interactions also between uncultivated species, which represent the majority [65,66]. Inference of co-occurrence networks is a computational method able to detect potential interactions between microbes based on their relative abundances in the samples [36]. This method is increasingly used to characterize the plant-associated microbiota, for example, in the rhizosphere [61,67,68,69] or in the pollen habitat [70]. Here we showed that *X. fastidiosa* is potentially interacting with several species of bacteria and fungi in the leaf endosphere of *Xf*-infected “Leccino”. Although we were particularly interested in negative correlations, which could indicate a direct inhibition of *X. fastidiosa*, the positive correlations could point to beneficial species that grow together with the pathogen (therefore being positively correlated) but at the same time modify its metabolism and reduce its pathogenicity. To perform this analysis we used only the endophytic samples from “Leccino” (and excluded ones of “Cellina di Nardò”) for two reasons: First, *X. fastidiosa* was consistently detected only in “Leccino” (Figure 1A; Figure S2); second, the microbiota of *Xf*-infected “Cellina di Nardò” was so different, due to the dysbiosis, that the network would have been strongly biased by the “habitat filtering” effect [36], which would make network interpretation impossible [71]. On the other hand, as a consequence, the number of samples used for the co-occurrence analysis in our work was relatively low, which is a critical point in co-occurrence network inference [71]. In the future, a wider analysis of the endophytic leaf microbiota from *Xf*-infected “Leccino” trees should be done, to perform a more robust co-occurrence correlation analysis and confirm our results. 

“Cellina di Nardò” *Xf*-infected samples in our study showed a low presence of *X. fastidiosa*. This was probably due to the fact that we sampled plants at a very advanced disease state, when the pathogen could become very erratic [72]. The putative lower amount of *X. fastidiosa* observed in 2018 in susceptible cultivar compared to resistant one should be likely due to the progression of symptoms, which became very severe in “Cellina di Nardò” trees in 2018, causing an adverse habitat for the pathogen. Moreover, the advanced state of disease in the *Xf*-infected “Cellina di Nardò” plants might have had an effect on the whole microbiome. However, to analyze such plants was a strategic decision because we aimed to have certainly *Xf*-infected olive trees as control to be compared to the resistant “Leccino” trees, which are definitively the subject of this work. To assess the actual effect of the disease status on the whole microbiome, an analysis of susceptible olive cultivars at different disease stages will be necessary, in order to investigate the dynamic of the dysbiosis and to link it to the general infection status of the tree. 

## 4. Methods

### 4.1. Sample Collection 

Olive trees of cultivars “Leccino” and “Cellina di Nardò” were sampled in spring 2016–2018 in productive orchards located close to the town of Lecce in Apulia (Southern Italy) (Parco Naturale Regionale Bosco e Paludi di Rauccio, 40°27′24.0″ N–18°09′43.8″ E, Apulia, Italy). 

Olive trees showing symptoms of OQDS were grown in neighboring “Leccino” and “Cellina di Nardò” orchards. Asymptomatic plants were grown in neighboring orchards of both cultivars, at a distance of about 3 km from the symptomatic plants’ orchards. The two sampling areas lie on a flat region without dividing or surrounding orographic elements (hills, rivers, etc.), with an even climate (Table S5). Physicochemical soil parameters of the two sampling areas were performed, and resulted largely similar (Table S6). Plants to be sampled were selected according to similar age (25–35 years), comparable disease status, same agronomic practices in the last 3 years (including pest control, according to both, recommendations for protected areas of the region [73] and phytosanitary treatments (EU Decision 2015/789).

The presence of *X. fastidiosa* was assessed by real-time PCR [74] in 2016–2018 (see section below). The selected plants were monitored for some of the most common pathogens in addition to *X. fastidiosa,* checking for symptoms caused by natural infection of *Spilocaea oleaginea* and *Pseudomonas savastanoi* pv. *savastanoi* during the 12 months before sampling. According to [72], the presence of symptoms of olive peacock spot and olive knot were scored singularly using a severity scale (0 = symptomless; 1 = symptoms on few branches (≤5); 2 = symptoms on several branches (>5); and 3 = symptoms uniformly distributed throughout the canopy). In addition, diagnostic tests (real-time PCR) were carried out on leaves or woody section, according to protocols reported in the literature, for *Botryosphaeria dothidea* [75], *Colletotrichum* spp., *C. acutatum* and *C. gloeosporioides* [76], *Diplodia seriata* [77], *Phaeomoniella chlamydospore* [78], *Phaeoacremonium aleophilum,* and *P. parasiticum* [79,80], *Phytophthora* spp. [81], and *Verticillium dahliae* [82].

To carry out the endophytic analysis on homogeneous trees, both *X. fastidiosa* positive and negative plants (5 plants per cultivar/infection status) were selected according to lower severity (= 1) for *P. savastanoi* pv. *savastanoi* (plants without symptoms of olive knot were not available in the area) and negative response to diagnostic test for the other pathogens (Table S7). However, *P. savastanoi* pv. *savastanoi* is not a systemic pathogen; thus the wood and leaves sampling for endophytic analysis were carried out on branches without olive knot symptoms in order to avoid interactions with *X. fastidiosa*.

Each biological replicate consisted of pooled branches or leaves collected from a single tree. In detail, every branches’ sample consisted of cross-sections without bark of pooled pieces (0.5 cm length), while each leaf sample consisted of a pool of 25 leaves. 

Samples were named according to the following identification code: first letter = cultivar (L or C, i.e., “Leccino” or “Cellina di Nardò”); second letter = infection status (I or H, i.e., *Xf*-infected or -uninfected); third letter = habitat (B or L, i.e., Branches or Leaves). A progressive number from 1 to 5 was added to identify the replicate. The prefix “Endo” was added to the samples that were subsequently surface-sterilized, to analyze the endophytic microbiota only. Examples: Endo-LHB-1 = the first replicate of “Leccino” *Xf*-uninfected branches, endophytes; CIL-2 = the second replicate of “Cellina di Nardò” *Xf*-leaves, total microbiota (epiphytes + endophytes). In total, 80 samples were analyzed: 2 cultivars*2 infection status (*Xf*-infected vs. -uninfected)*2 plant habitats (branches or leaves)*2 community type (epiphytic + endophytic vs. endophytic only)*5 replicates, according to a full-factorial scheme.

For the surface sterilization, the following protocol was used: 75% EtOH for 1 min, 4% NaOCl for 5 min, and 75% EtOH for 30 sec.

### 4.2. DNA Extraction, PCR, and Illumina Sequencing of 16S rRNA Gene and ITS2 Amplicon Libraries

Plant tissue from each sample (approximately 1 g of leaf petioles and branches) was transferred into an extraction bag (BIOREBA, Switzerland) and 4 mL of extraction buffer (0.2 M of Tris–HCl pH 9, 0.4 M of LiCl, and 25 mM of EDTA) were added. Sample homogenization was performed by using a semi-automatic homogenizer (Homex 6, BIOREBA, Switzerland) at 50% maximum speed. DNA extraction was performed according to Edwards et al. [83] with some modifications. In this protocol, the DNA solution is first extracted with a phenol/chloroform/isoamyl alcohol (25:24:1) mixture, to remove protein contaminants, and then precipitated with 100% isopropanol. 

The isolated DNA was used as template for *X. fastidiosa* detection by TaqMan real-time PCR protocol with XF-F/R primers and XF-P probe [74]. Reactions were performed in a real-time thermal cycler (ABI PRISM 7900HT, Applied Biosystems, USA). Each reaction consisted of 5 μL from a 20 ng μL^−1^ dilution of DNA extracted from 1 g of leaf petioles or branches, 12.5 μL of Master Mix (Applied Biosystems), 400 nM of forward and reverse primers, 200 nM of TaqMan probe, and ultrapure DNase/RNase-free water (Carlo Erba Reagents S.r.l., Italy) in a total volume of 25 μL. The cycling conditions were as follows: an initial denaturation step at 95 °C for 10 min, followed by 40 cycles of 95 °C for 15 s and 60 °C for 1 min, with the final dissociation at 95 °C for 15 s, 60 °C for 30 s, and 95 °C for 15 s. All symptomatic and asymptomatic plants were singularly tested each year (2016–2018). Trees were considered *Xf*-uninfected when leaf samples were negative to every *X. fastidiosa* assay carried out in 2016-2018. In regard to *Xf*-infected plants, *X. fastidiosa* concentration from “Leccino” and “Cellina di Nardò” samples were inferred by the standard calibration curve, using Cqs from qPCR as described by [84] (Table S7). 

The extracted DNA was also used as the template to perform a two-step amplification protocol in which the core PCR primer and the adaptors CS1 (forward) and CS2 (reverse) were included in a single oligonucleotide at the 5′ end of each primer to allow multiplexing with the Fluidigm Access Array System (Fluidigm, South San Francisco, CA, USA). The oligonucleotide sequences were (core PCR primer in bold) 5.8S-Fun (5′-ACACTGACGACATGGTTCTACA-**AACTTTYRRCAAYGGATCWCT**-3′) and ITS4-Fun (5′-TACGGTAGCAGAGACTTGGTCT-**CCTCCGCTTATTGATATGCTTAART**-3′) [85] for fungi, 341F (5′-ACACTGACGACATGGTTCTACA-**CCTAYGGGDBGCWSCAG**-3′) and 806R (5′-TACGGTAGCAGAGACTTGGTCT-**GGACTACNVGGGTHTCTAAT**-3′) for prokaryotes [86]. A mixture of peptide nucleotide acid (PNA) blockers oligos (PNA Bio Inc., Thousand Oaks, CA, USA) targeted at plant mitochondrial and plastidic genomes was added, to increase the fraction of bacterial sequences, reduce the PCR-bias, and thus result in more accurate sequencing [86,87].

Briefly, the PCR was carried out in 25 µL of reaction mixture, using 50 ng of DNA, 12.5 µL 2× PCR master mix (EmeraldAmp^®^ GT PCR Master Mix, Takara Bio, Inc., Japan), 1.0 µL FWD primer (0.2 µM), 1.0 µL REV primer (0.2 µM), 1.25 mPNA blocker (0.25 µM), 1.25 pPNA blocker (0.25 µM), and ultrapure DNase/RNase-free water (Carlo Erba Reagents S.r.l., Italy) to final volume of 25 µL. PCR was performed in 2720 Thermal Cycler (Applied Biosystems, USA), using the following program: initial denaturing at 94 °C for 3 min, followed by 30 or 35 cycles (for prokaryota or fungi) of denaturation at 94 °C for 15 s, PNA clamping at 75 °C for 10 s, primer annealing at 58 °C for 10 s (both primer pairs), extension at 72 °C for 60 s, followed by a final extension at 72 °C for 10 min and by cooldown to 4 °C. 

The integrity and quality of the PCR products were checked on an agarose gel. PCR was repeated three times per sample, replicates were pooled [88] and sent for sequencing on an Illumina MiSeq platform (v3 chemistry) at the Génome Québec Innovation Center at the McGill University (Montréal, Canada).

The sequence datasets generated during and analyzed during the current study are available in the European Nucleotide Archive (ENA) repository (www.ebi.ac.uk/ena) under the project number PRJEB32050.

### 4.3. Bioinformatic Analysis of the Sequences

Illumina sequencing data were analyzed with QIIME 1.9 [89]. After joining paired ends with the “fastq-join” method [90], a quality filtering was applied (quality threshold: 25). Length boundaries of 200–1000 nucleotides were used to eliminate short sequences, such as primer dimers, as well as overly long sequences, such as wrongly merged reads. To extract fungal ITS reads, ITSx v.1.0.11 was used [91].

Chimeric sequences were removed using Vsearch [92]. Operational taxonomic units (OTUs) were generated at 97% sequence similarity by using the sumaclust method, which was shown to be more accurate than other clustering methods [93], and were identified, after alignment, using the uclust method and the databases SILVA, release 132 [94], for prokaryotes and UNITE v.7.2, release_s_01.12.2017 [95], for fungi. Unidentified and contaminant (plastidic and mitochondrial for Prokaryotes, Metazoa for Fungi) OTUs were removed, using the QIIME script “filter_taxa_from_otu_table.py”, as well as OTUs with less than 10 reads, to reduce noise. Samples with a low number of reads (threshold: 11,000 and 4000 reads for 16s rRNA gene and ITS2 dataset, respectively) were eliminated to optimize the per-sample coverage. Alpha- and beta-diversity metrics were calculated on rarefied datasets normalized to an even sequencing depth per sample: this was 11,950 reads for 16S rRNA gene dataset and 4850 reads for ITS2 dataset, when including all kept samples. When the analysis was performed on subsamples of the datasets, the normalization was always adapted to the sample with lowest number of sequences, in order to optimize robustness and reliability of the statistical analyses. For example, when analyzing only the *Xf*-infected Leaf samples, the rarefaction values were the 18,450 and 7180 reads per sample for the 16S and the ITS2 dataset, respectively.

Statistical comparison of alpha diversity between samples was performed with SPSS version 20 (IBM Corporation, USA), using factorial ANOVA and Duncan’s post hoc test at alpha-level = 0.05. Normality of distribution and heteroscedasticity were tested with the Shapiro–Wilk and the Levene’s tests, respectively. The factors considered were as follows: “Infection status” (2 levels, *Xf*-infected and -uninfected), “Cultivar” (2 levels, “Leccino” and “Cellina di Nardò”), “Habitat” (2 levels, branches and leaves), and their interactions. The “Type of microbiota” (total community, epiphytes + endophytes, or endophytes only) was also tested, however, only for its main effect, since the endophytes are a subpopulation of the total microbiota and their diversity is obviously lower. Beta-diversity (distance between samples’ microbiome structures) was computed separately on total community and endophytes, by using Bray–Curtis distances; the effects of the investigated factors were statistically assessed with the Adonis test [96]. The script “group_significance.py” was used to perform g-test of independence as implemented in QIIME. Beta-diversity plots (Principal Component Analysis, PCoA) were visualized by Emperor [97]. The full list of scripts used for each QIIME pipeline is available upon request. The software STAMP [98] was used to assess significantly different taxa between sample groups, using the nonparametric White’s test [99] for two-group comparisons (“Leccino” vs. “Cellina di Nardò”) and the Kruskal–Wallis test [100] for four-group comparisons (“Leccino” vs. “Cellina di Nardò” × *Xf*-infected vs. -uninfected) at alpha-level = 0.05. All *p*-significance values were corrected for multiple testing using the false discovery rate (FDR) adjustment according to Benjamini-Hochberg [101].

The software Explicet [102] was used to generate taxonomy bar plots, Bray–Curtis distances heat-maps and rarefaction curves. Extended error plots and the Box plots were directly exported from STAMP. Adobe Photoshop CS6 (Adobe Systems Inc., USA) was used to assemble and label the final figures.

### 4.4. Correlation of Co–Occurrence Patterns and Network Analysis

To identify significant patterns of correlated bacteria in the *Xf*-infected leaves of “Leccino” trees, the fungal and bacterial OTUs with at least 30 total reads and occurring in at least 3 samples were subjected to a co-occurrence analysis, using the software CoNet [103], which is available as add-on in Cytoscape 3.7 [104]. Non-rarefied, absolute abundance dataset was used, as recommended to avoid the false-positive issues when using relative abundance data [70]. A preliminary check of the dataset indicated a total of 1378 possible interactions. Therefore, we set a threshold of 650 top and bottom edges in the network (edge = correlation between two OTUs). Then, we computed a distribution of pairwise scores for each of the following similarity measures: Bray–Curtis distances, Kullback–Leibler dissimilarity, and Pearson and Spearman correlations. For each measure, 100 permutations were generated (with renormalization for correlation measures and row-shuffling) at 100 bootstrap-resampling scores. Unstable edges (with score outside the 2.5–97.5 percentiles of the bootstraps’ distribution) were removed. The four measure-specific *p*-values were then merged by using Brown’s method; variances were pooled by combining the *p*-values of permutations and bootstraps. After FDR-correction, using the Benjamini–Hochberg method, edges with a *p*-value below 0.05 were kept. Considering that the distance measures (Bray–Curtis and Kullback–Leibler) are sensitive to outliers and robust to compositionality, while correlation measures (Pearson and Spearman) are biased by compositionality and robust to outliers [103], only edges supported by at least three similarity measures were retained. The network layout was arranged using the “edge-forced spring embedded” algorithm [105], additionally weighted by *p*-values; this method draws unbiased networks with interconnected nodes close each other and single-linked ones placed apart [106]. “Hub” nodes, representing taxa with disproportionately large effect in shaping the microbial network [107], were identified with the software cytoHubba [108], based on degree (number of connections) and betweenness centrality values [69,107].

Legends of the network were created with the Cytoscape-App “Legend creator” (http://apps.cytoscape.org/apps/legendcreator). Adobe Photoshop CS6 (Adobe Systems Inc., USA) was used to assemble and label the final figures.

## 5. Conclusions

In this work, we performed a large-scale observational study of the olive tree microbiome in a *X. fastidiosa*-resistant and a *X. fastidiosa*-susceptible cultivar in the Salento region, an area where a severe *X. fastidiosa* outbreak has been taking place since 2013. Our results highlighted the stability of the endophytic bacterial microbiota in the leaves of *Xf*-infected “Leccino” cultivar (*X. fastidiosa*-resistant), in contrast with the strong dysbiosis observed in the *X. fastidiosa*-susceptible cultivar “Cellina di Nardò”.

The microbiota of “Leccino” trees are also more diverse and include cultivar-specific bacterial taxa that appear to interact, directly or indirectly, with *X. fastidiosa*.

The maintenance of a healthy microbiota and the presence of cultivar-specific microbes might support the resistance of “Leccino” to *X. fastidiosa* infection. Our results represent a first step toward the identification of microbial species or consortia with potential biotechnological applications for the biological treatment of the Olive Quick Decline Syndrome in Salento. Our data could be used to perform a targeted isolation of candidate microorganisms of interest to be tested against *X. fastidiosa* under experimental conditions.

## Figures and Tables

**Table 1:** Adonis tests based on the Bray–Curtis distances between samples of the olive-associated bacterial and fungal microbiota. The test was performed separately for total microbiota (epiphytes + endophytes) and for endophytes only. Significant values (*p* < 0.05) are indicated in bold. *Xf* = *Xylella fastidiosa*.

		*p* Values			
		Bacteria		Fungi	
*Factor*	*Subject*	*Epiphytes + Endophytes*	*Endo-phytes*	*Epiphytes + Endophytes*	*Endo-Phytes*
Infection status	All samples	**0.001**	**0.001**	**0.024**	0.786
Infection status	“Cellina di Nardò”	**0.001**	**0.001**	0.069	0.446
Infection status	“Leccino”	0.067	0.069	**0.001**	0.184
Infection status	“Cellina di Nardò” Branches	**0.001**	**0.028**	**0.001**	0.369
Infection status	“Leccino” Branches	**0.007**	**0.012**	**0.001**	**0.0014**
Infection status	“Cellina di Nardò” Leaves	**0.001**	**0.001**	**0.001**	0.902
Infection status	“Leccino” Leaves	**0.035**	0.429	**0.006**	0.114
Cultivar	All samples	**0.002**	**0.001**	**0.004**	0.081
Cultivar	*Xf*-infected	**0.001**	**0.001**	**0.028**	0.05
Cultivar	*Xf*-uninfected	**0.001**	**0.040**	**0.001**	0.276
Cultivar	*Xf*-infected Branches	**0.001**	**0.009**	**0.001**	0.055
Cultivar	*Xf*-infected Leaves	**0.001**	**0.013**	**0.001**	0.122
Cultivar	*Xf*-uninfected Branches	**0.011**	0.243	**0.001**	**0.0014**
Cultivar	*Xf*-uninfected Leaves	**0.001**	0.101	**0.001**	0.32
Habitat	All samples	**0.014**	0.483	**0.001**	0.205
Habitat	“Cellina di Nardò”	0.874	0.723	**0.001**	0.264
Habitat	“Leccino”	**0.001**	0.061	**0.001**	0.256
Habitat	*Xf*-infected	0.138	0.368	**0.001**	0.207
Habitat	*Xf*-uninfected	**0.020**	0.763	**0.001**	0.271

## Supplementary Materials

The following are available online at https://www.mdpi.com/2076-0817/9/1/35/s1. Table S1: Number of high-quality reads obtained by Illumina sequencing. Table S2: Number of replicates per sample type analyzed in this work. Table S3 (provided as excel file): Bacterial OTU tables. Table S4 (provided as excel file): Fungal OTU tables. Table S5: Meteorological data records of the last three years in the area in which *Xylella fastidiosa*-infected or *Xylella fastidiosa*-uninfected samples were collected. Table S6: Physico-chemical soil analysis of sampling areas in which *Xylella fastidiosa*-infected or *Xylella fastidiosa*-uninfected samples were collected compared to typical reference values. Table S7: Analysis of pathogen species in the trees selected for this work. Figure S1: Rarefaction curves. Figure S2: Relative abundance of *Xylella* in the analyzed samples. Figure S3: Per-sample beta-diversity metrics of bacteria. Figure S4: Per-group beta-diversity metrics of bacteria and fungi. Figure S5: Per-sample beta-diversity metrics of fungi. Figure S6: Original co-occurrence network. Figure S7: Distribution of the OTU identified as *Planomicrobium* among sample groups.
