# Supplementary material for: The Xylella fastidiosa-Resistant Olive Cultivar “Leccino” Has Stable Endophytic Microbiota during the Olive Quick Decline Syndrome (OQDS)
Source: Pathogens. 2019 Dec 31;9(1):35. doi: 10.3390/pathogens9010035 (PMC7168594; doi:10.3390/pathogens9010035)
Supplement: Supplementary file 1 [file pathogens-09-00035-s001.zip › Supplementary materials_corrected proofs.pdf]

The *Xylella fastidiosa*-resistant olive cultivar "Leccino" has stable endophytic microbiota during the Olive Quick Decline Syndrome (OQDS)

Marzia Vergine, Joana B. Meyer, Massimiliano Cardinale*, Erika Sabella, Martin Hartmann, Paolo Cherubini, Luigi De Bellis and Andrea Luvisi

*corresponding author: massimiliano.cardinale@unisalento.it

Supplementary material

Table S1: **Number of high-quality reads obtained by Illumina sequencing**

Table S2: **Number of replicates per sample type analyzed in this work**

Table S3 (provided as excel file): **Bacterial OTU tables**

Table S4 (provided as excel file): **Fungal OTU tables**

Table S5: **Meteorological data records of the last three years in the area in which *Xylella fastidiosa*-infected or *Xylella fastidiosa*-uninfected samples were collected**

Table S6: **Physico-chemical soil analysis of sampling areas in which *Xylella fastidiosa*-infected or *Xylella fastidiosa*-uninfected samples were collected, compared to typical reference values**

Table S7: **Analysis of pathogen species in the trees selected for this work**

Figure S1: **Rarefaction curves**

Figure S2: **Relative abundance of *Xylella* in the analyzed samples**

Figure S3: **Per-sample beta-diversity metrics of bacteria**

Figure S4: **Per-group beta-diversity metrics of bacteria and fungi**

Figure S5: **Per-sample beta-diversity metrics of fungi**

Figure S6: **Original co-occurrence network**

Figure S7: **Distribution of the OTU identified as *Planomicrobium* among sample groups**

Table S1 Number of high-quality reads obtained by Illumina sequencing. Per-sample number of reads obtained by Illumina sequencing of both, prokaryotic 16S rRNA genes and fungal ITS2, gene libraries, after quality/length filtering and removal of chimeric, unassigned and contaminant (plastidic or mitochondrial) sequences. Sample naming: refer to the Methods section in the main text, paragraph "*Sample collection*". Samples are sorted by sample type and numbered according to the replicate. Some replicates were excluded due to a low number of reads (threshold: 11000 reads for 16S rRNA genes, 4000 reads for ITS2; see text and Tab. S3).

**16S rRNA gene sequencing**		**ITS2 sequencing**	
**Sample**	**N. of reads**	**Sample**	**N. of reads**
Endo-CIL 1	112356	Endo-CIL 2	9246
Endo-CIL 2	121289	Endo-CIL 3	7188
Endo-CIL 3	18476	Endo-CIL 4	18132
Endo-CIL 4	79479	Endo-CIL 5	16620
Endo-CIL 5	69029	Endo-CHL 1	12552
Endo-CHL 1	13045	Endo-CHL 2	44977
Endo-CHL 2	34379	Endo-CHL 3	12931
Endo-CHL 3	26405	Endo-CHL 5	12242
Endo-CHL 4	19440	CIL 1	81686
CIL 1	73622	CIL 2	83837
CIL 2	61550	CIL 3	11769
CIL 3	96191	CIL 4	46426
CIL 4	90289	CIL 5	52191
CIL 5	96217	CHL 1	47904
CHL 2	26039	CHL 2	57804
CHL 3	27928	CHL 3	69469
CHL 4	26415	CHL 4	60983
CHL 5	30342	CHL 5	34885
Endo-CIB 1	84338	Endo-CIB 1	10836
Endo-CIB 3	89591	Endo-CIB 2	12778
Endo-CIB 4	78432	Endo-CIB 3	17247
Endo-CIB 5	87632	Endo-CIB 4	10080
Endo-CHB 2	27132	Endo-CIB 5	6746
Endo-CHB 3	18775	Endo-CHB 2	9150
Endo-CHB 4	46662	Endo-CHB 3	7784
CIB 1	110967	Endo-CHB 4	18894
CIB 2	95852	CIB 1	20336
CIB 3	60886	CIB 2	19659
CIB 4	60282	CIB 3	24547
CIB 5	62723	CIB 4	15012
CHB 1	29585	CIB 5	17274
CHB 2	34544	CHB 1	4856
CHB 3	29187	CHB 2	22279
CHB 4	11970	CHB 3	21446

CHB 5	33527
Endo-LIL 1	25383
Endo-LIL 2	29694
Endo-LIL 3	36180
Endo-LIL 4	20793
Endo-LIL 5	26235
Endo-LHL 2	70769
Endo-LHL 3	21676
Endo-LHL 4	47129
Endo-LHL 5	58929
LIL 1	30069
LIL 2	51113
LIL 3	61505
LIL 4	21722
LIL 5	55012
LHL 2	58233
LHL 3	79180
LHL 4	56178
LHL 5	47390
Endo-LIB 1	21039
Endo-LIB 2	52817
Endo-LIB 3	45969
Endo-LIB 4	46134
Endo-LIB 5	65607
Endo-LHB 1	25693
Endo-LHB 2	40770
Endo-LHB 3	66825
Endo-LHB 4	79481
Endo-LHB 5	65927
LIB 1	18461
LIB 2	46065
LIB 3	58988
LIB 4	35132
LIB 5	53413
LHB 1	38536
LHB 2	93423
LHB 3	42660
LHB 4	104971
LHB 5	15917

Endo-LIL 1	7619
Endo-LIL 2	31213
Endo-LIL 3	17883
Endo-LIL 4	29482
Endo-LIL 5	17723
Endo-LHL 2	24772
Endo-LHL 3	25741
Endo-LHL 4	11492
LIL 1	81520
LIL 2	92764
LIL 3	77072
LIL 4	86801
LIL 5	91974
LHL 2	84869
LHL 3	104659
LHL 4	44942
LHL 5	71582
Endo-LIB 1	5512
Endo-LIB 4	60490
Endo-LIB 5	45718
Endo-LHB 3	26328
Endo-LHB 4	42762
Endo-LHB 5	40658
LIB 1	52098
LIB 2	22758
LIB 3	32118
LIB 4	28778
LIB 5	7888
LHB 1	35636
LHB 2	64908
LHB 3	91617
LHB 4	75503
LHB 5	12109

Table S2 Number of replicates per sample type analyzed in this work. Number of biological replicates per sample type remained after removing samples that did not give a PCR product and samples with a low number of reads. The original sampling was n= 5 for each sample type. *Xf*= *Xylella fastidiosa*.

**Sample type**	**N. of biological replicates**	
	**16S rRNA gene libraries**	**ITS2 libraries**
"Cellina di Nardò" *Xf*-uninfected Branches	5	3
"Cellina di Nardò" *Xf*-uninfected Branches Endophytes	3	3
"Cellina di Nardò" *Xf*-uninfected Leaves	4	5
"Cellina di Nardò" *Xf*-uninfected Leaves Endophytes	4	4
"Cellina di Nardò" *Xf*-infected Branches	5	5
"Cellina di Nardò" *Xf*-i nfected Branches Endophytes	4	5
"Cellina di Nardò"*Xf*-i nfected Leaves	5	5
"Cellina di Nardò" *Xf*-i nfected Leaves Endophytes	5	4
"Leccino" *Xf*-uninfected Branches	5	5
"Leccino" *Xf*-uninfected Branches Endophytes	5	3
"Leccino" *Xf*-uninfected Leaves	4	4
"Leccino" *Xf*-uninfected Leaves Endophytes	4	3
"Leccino" *Xf*-infected Branches	5	5
"Leccino" *Xf*-infected Branches Endophytes	5	3
"Leccino" *Xf*-infected Leaves	5	5
"Leccino" *Xf*-infected Leaves Endophytes	5	5
**TOTAL**	**73**	**67**

Table S3 (provided as excel file): **Bacterial OTU tables**

Table S4 (provided as excel file): **Fungal OTU tables**

Table S5: Meteorological data records of the last three years in the area in which *Xylella fastidiosa*-infected or *Xylella fastidiosa*-uninfected samples were collected. Data from the weather station "Lecce 163320" (latitude 40°24′86.71′′; longitude 18°13°70.95′′); (www.scia.isprambiente.it/wwwrootscia/Home_new_eng.html).

Meteorological parameter	Value
**Mean temperature (°C)**	
2016	17.2±6.7
2017	16.5±7.3
2018	17.3±6.7
**Mean maximum temperature (°C)**	
2016	22.6±7.2
2017	22.5±8.4
2018	22.4±7.3
**Mean minimum temperature (°C)**	
2016	11.8±6.6
2017	10.6±6.6
2018	12.2±6.5
**Annual precipitation (mm)**	
2016	525.4
2017	482.2
2018	1005.9
**Mean relative humidity (%)**	
2016	72.7
2017	75.0
2018	80.1

Table S6: **Physico-chemical soil analysis of sampling areas in which *Xylella fastidiosa*-infected or *Xylella fastidiosa*-uninfected samples were collected, compared to typical reference values.**

Physico-chemical parameter	*Xylellafastidiosa*-infected sampling area	*Xylella fastidiosa*-uninfected sampling area	Reference values*	Reference values for olive**
Texture (% w/w)				
Sand	76	77	-	
Lime	10	10	-	
Clay	14	13	-	
pH	9.11	7.90	5.5-8.50	
Electrical conductivity (μS cm^{-1})	1,329	1,085	600-2,000	
Total organic carbon (mg kg^{-1} dw)	13,773.88	12,527.69	9,000-13,600	
Organic matter (mg kg^{-1} dw)	23,737.00	21,589.39	12,000-30,000	
Free lime (g kg^{-1})	22.26	19.85	50-100	
Total lime (g kg^{-1})	212.83	185.00	50-200	
Nitrogen (mg kg^{-1} dw)	1,917.80	1,699.56	1,000-2,000	1,000 -5,000
Potassium [meq (100 g^{-1})]	1.24	0.64	0.27-0.39	0.20 - > 0.64
Calcium [meq (100 g^{-1})]	224.37	85.85	3-10	
Sodium [meq (100 g^{-1})]	2.93	0.55	0-2	
Magnesium [meq (100 g^{-1})]	11.28	2.46	0.9-1.2	
Phosphorus (mg kg^{-1})	222.00	73.60	12-20	10 - > 40
Copper (mg kg^{-1} dw)	16.69	31.62	2-10	
Zinc (mg kg^{-1} dw)	2.47	4.64	2-10	
Iron (mg kg^{-1} dw)	658.17	250.94	60-20	
Manganese (mg kg^{-1} dw)	143.21	172.95	4-20	

* Vv.A.a.; Appendice: specifiche delle proprietà e qualità dei suoli. In *Metodi di valutazione dei suoli e delle terre,* Costantini, E.A.C., Ed.; Cantagalli: Siena, Italy, 2006; pp. 912-929.

** Vitagliano C.; Sebastiani L. Impianto. In *Olea trattato di olivicoltura*, 1st ed.; Fiorino, P., Ed.; Edagricole: Bologna, Italy, 2003; pp 195-209.

Table S7 Analysis of pathogen species in the trees selected for this work. Sampling was carried out in 2018, except for *Xylella fastidiosa*, which was assayed in 2016-2018. Pathogen data was expressed as symptoms presence (*Spilocaea oleaginea*, *Pseudomonas savastanoi* pv. *savastanoi*), C_t of quantitative-PCR assay (*Xylella fastidiosa*), or positive/negative (+/-) response to test (other pathogens). *Xf*= *Xylella fastidiosa*. *X. fastidiosa* concentration from samples were inferred by the standard calibration curve using Cqs from qPCR, or positive/negative (+/-) response to test (other pathogens).

**Pathogen species**	***Olea europaea* cv. "Leccino"**		***Olea europaea* cv. "Cellina di Nardò"**	
	***Xf*-infected (5 trees)**	***Xf*-uninfected (5 trees)**	***Xf*-infected (5 trees)**	***Xf*-uninfected (5 trees)**
Spilocaea oleaginea	Asymptomatic	Asymptomatic	Asymptomatic	Asymptomatic
Pseudomonas savastanoi pv. *savastanoi*	Symptomatic	Symptomatic	Symptomatic	Symptomatic
Botryosphaeria dothidea	-	-	-	-
Colletotrichum spp.	-	-	-	-
Colletotrichum acutatum	-	-	-	-
Colletotrichum gloeosporioides	-	-	-	-
Diplodia seriata	-	-	-	-
Phaeomoniella chlamydospora	-	-	-	-
Phaeoacremonium aleophilum	-	-	-	-
Phaeoacremonium parasiticum	-	-	-	-
Phytophthora spp.	-	-	-	-
Verticillium dahliae	-	-	-	-
Xylella fastidiosa (2016 sampling)	10^3-10^4	NA	10^6-10^7	NA
Xylella fastidiosa (2017 sampling)	10^4-10^5	NA	10^5-10^6	NA
Xylella fastidiosa (2018 sampling)	10^5-10^6	NA	10^4-10^5	NA

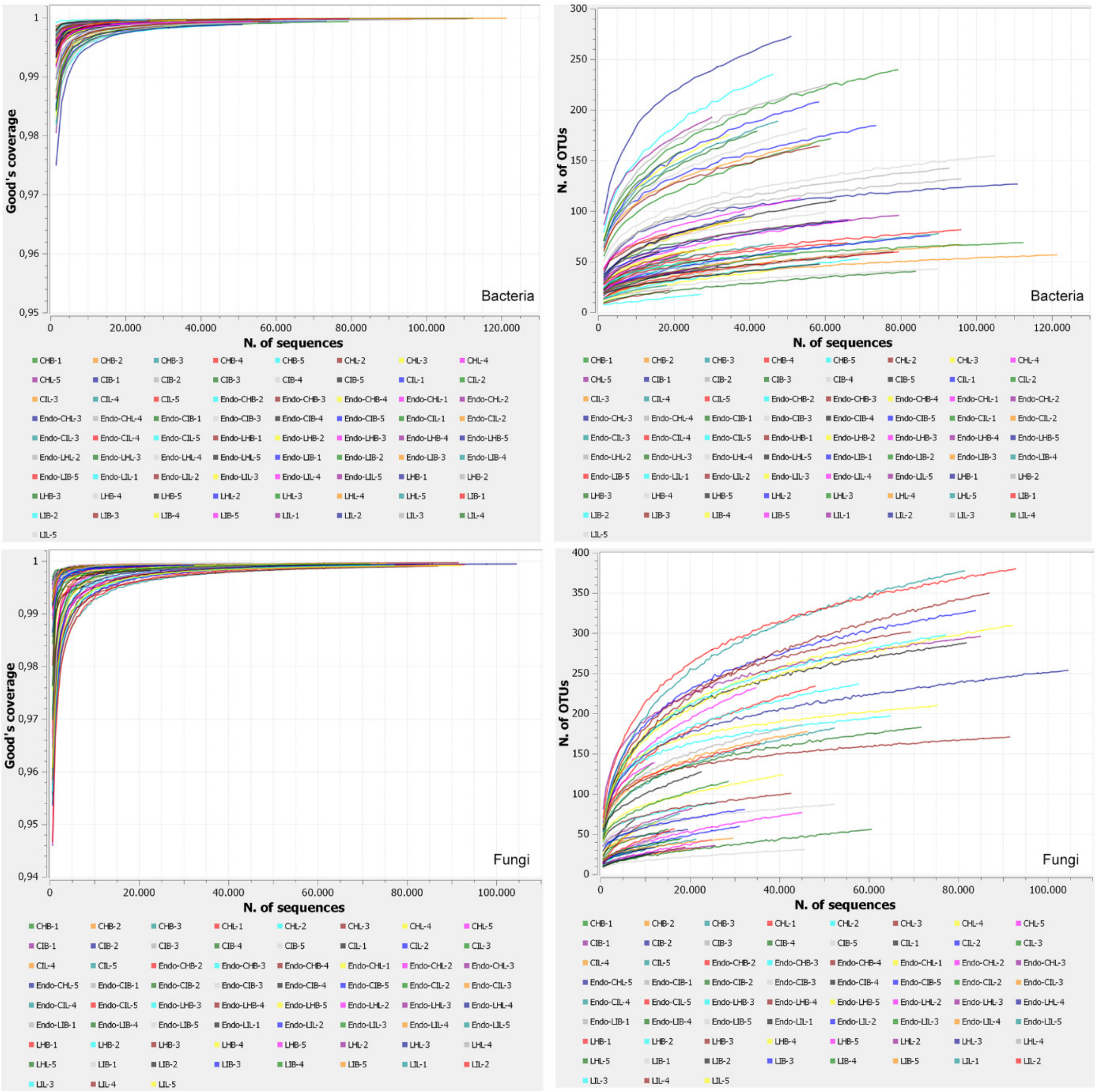

Fig. S1 Rarefaction curves. Good's coverage and cumulative number of OTUs per sample, for 16S rRNA gene libraries (upper plots) and ITS2 libraries (lower plots).

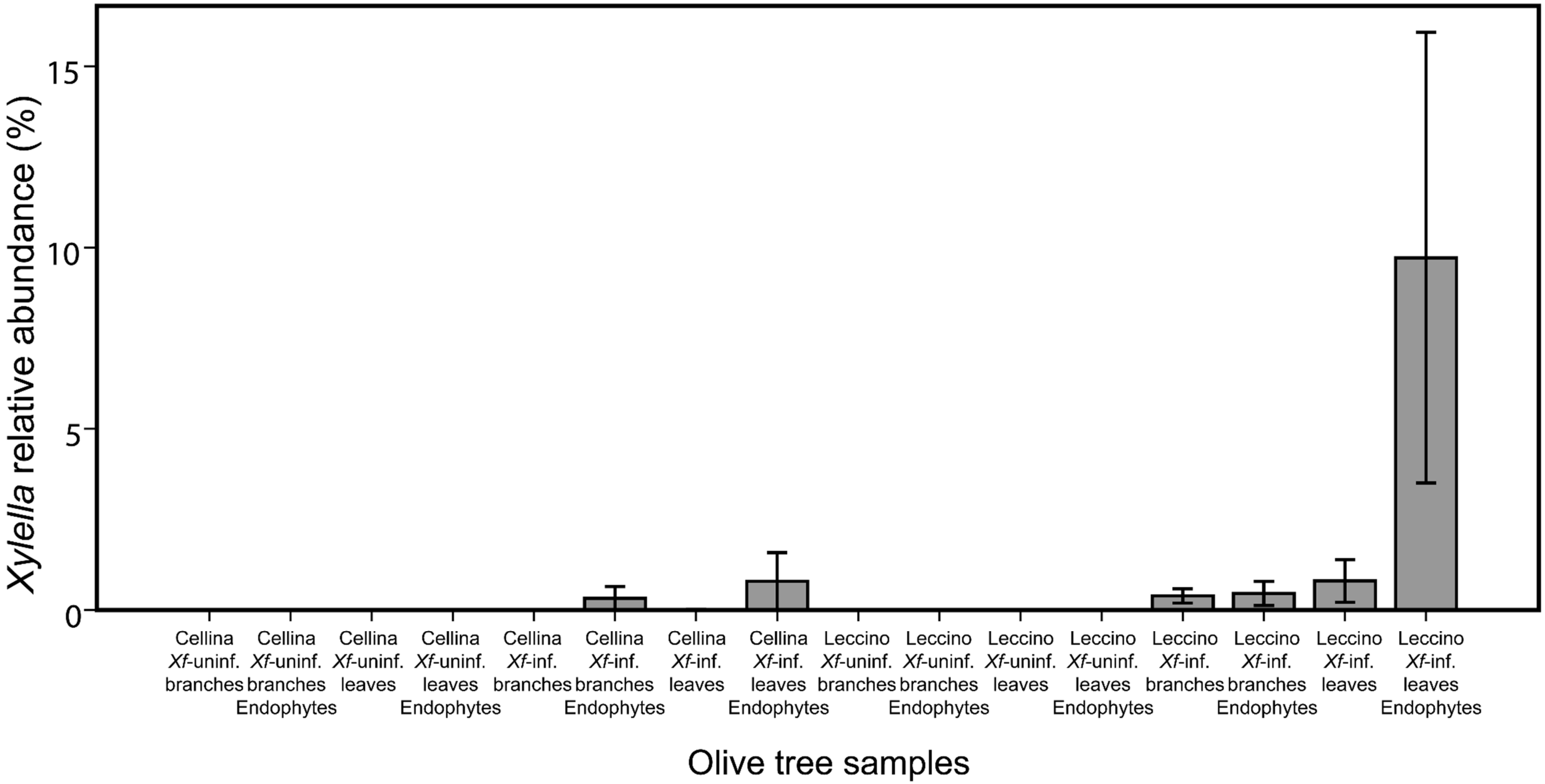

Fig. S2 Relative abundance of *Xylella* in the analyzed "Leccino" and "Cellina di Nardò" olive cultivars. Average relative abundance (± standard error), with respect to the total number of reads, of the genus *Xylella* in the sample groups. *Xf*-inf. = *Xylella fastidiosa*-infected samples; *Xf*-uninf. = *Xylella fastidiosa*-uninfected samples.

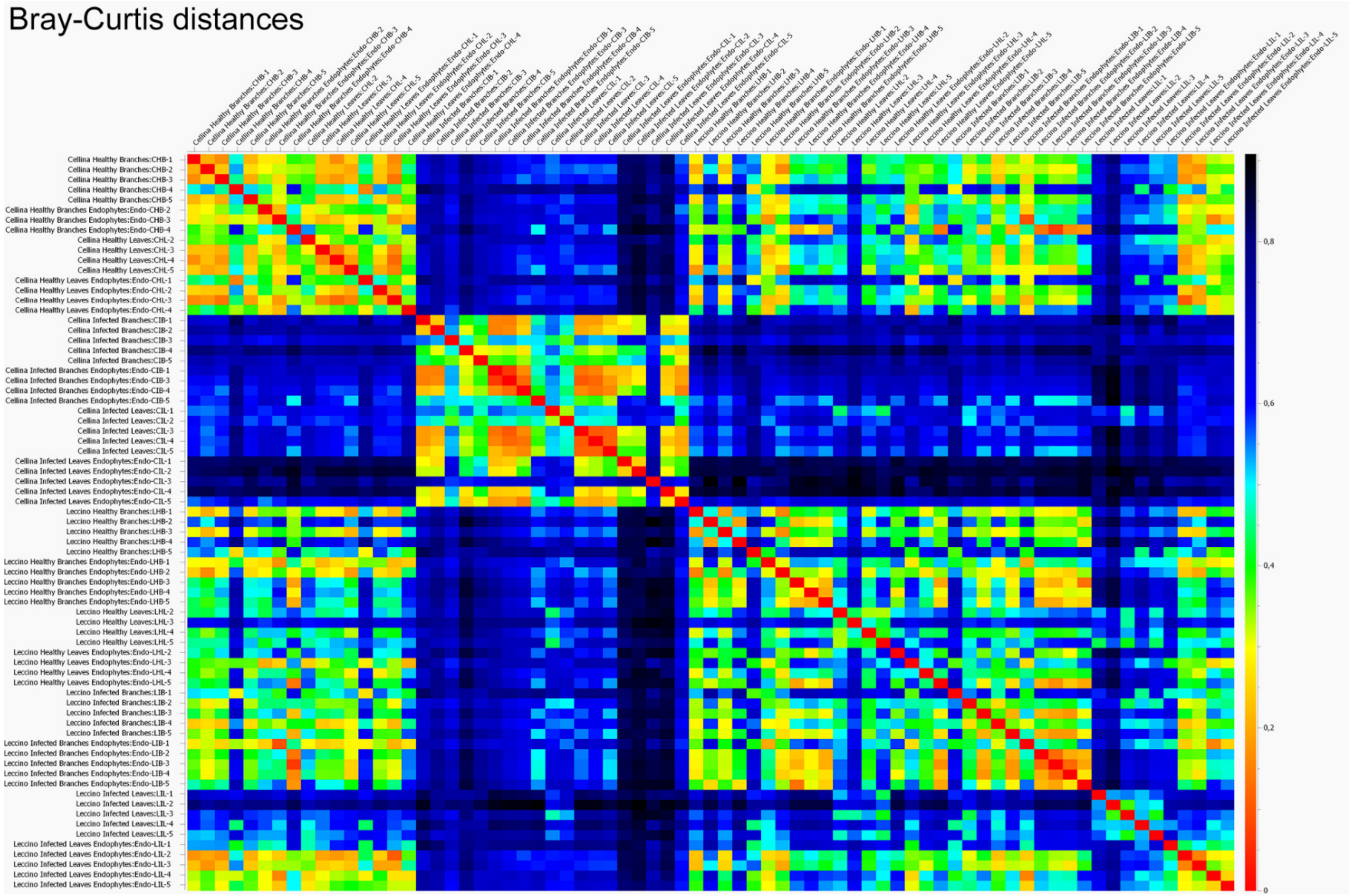

Fig. S3 Per-sample beta-diversity metrics of bacterial microbiota in the analyzed “Leccino” and “Cellina di Nardò” olive cultivars. Bray-Curtis distances matrix of the olive bacterial microbiota. Samples are ordered by cultivar, then by sample type. “Infected” = *Xylella fastidiosa*-infected samples; “Healthy” = *Xylella fastidiosa*-uninfected samples.

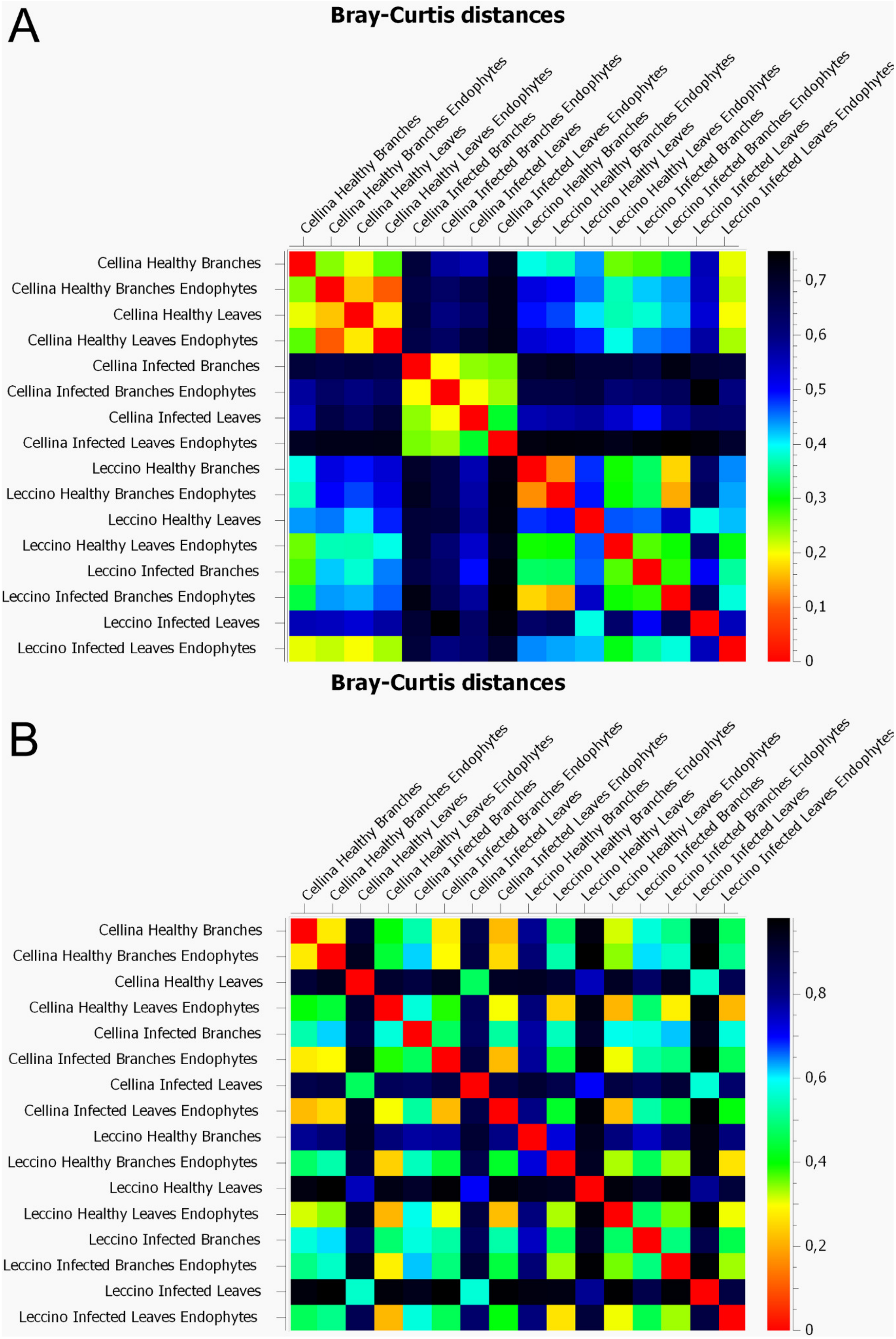

Fig. S4 Per-group beta-diversity metrics of bacterial and fungal microbiota in the analyzed "Leccino" and "Cellina di Nardò" olive cultivars. Bray-Curtis distances matrix of the olive bacterial (A) and fungal (B) microbiota. Sample groups are ordered by cultivar and grouped by sample type. "Infected" = *Xylella fastidiosa*-infected samples; "Healthy" = *Xylella fastidiosa*-uninfected samples.

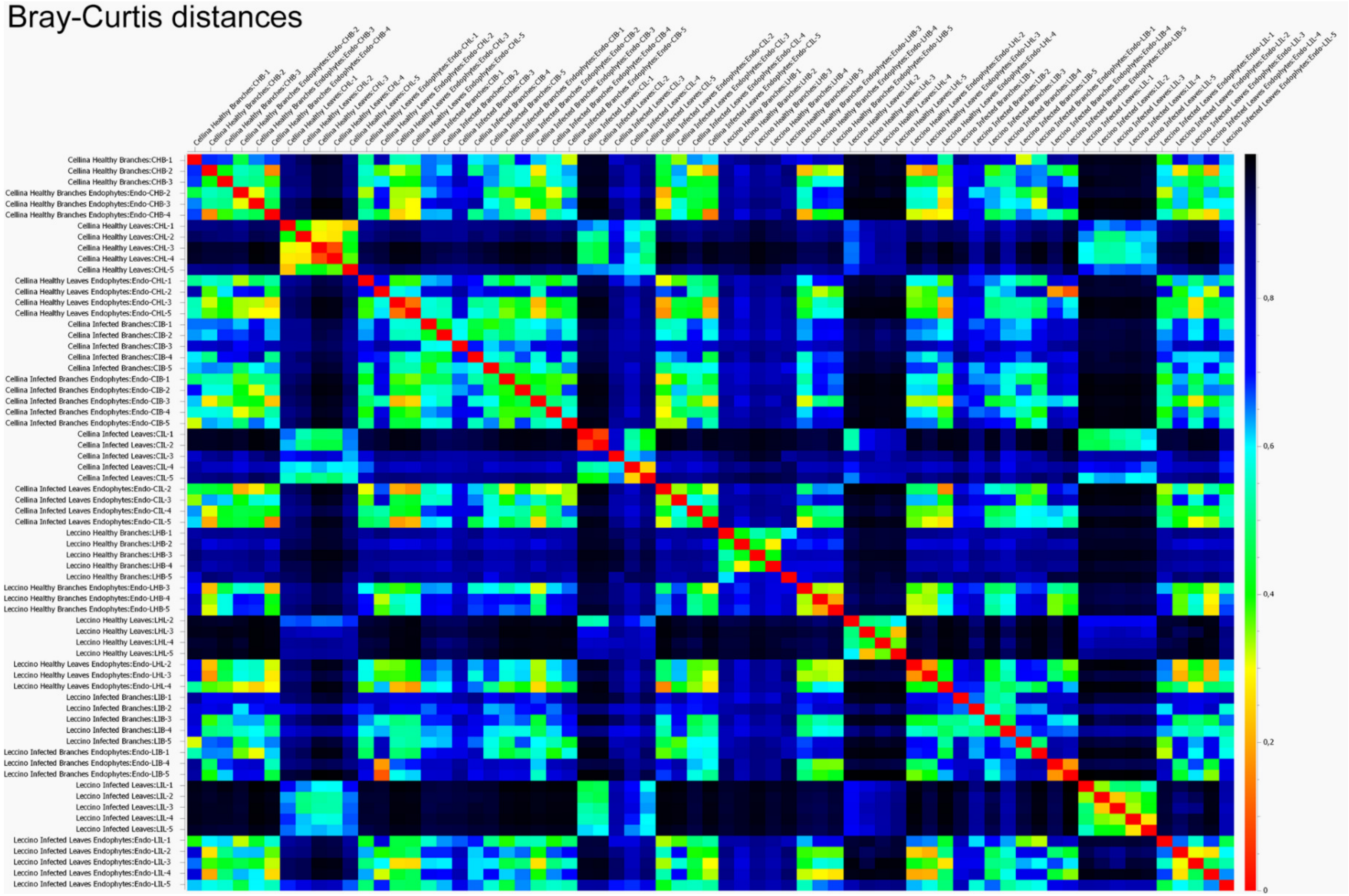

Fig. S5 Per-sample beta-diversity metrics of fungal microbiota in the analyzed "Leccino" and "Cellina di Nardò" olive cultivars. Bray- Curtis distances matrix of the olive fungal microbiota. Samples are ordered by cultivar, then by sample type. "Infected" = *Xylella fastidiosa*-infected samples; "Healthy" = *Xylella fastidiosa*-uninfected samples.

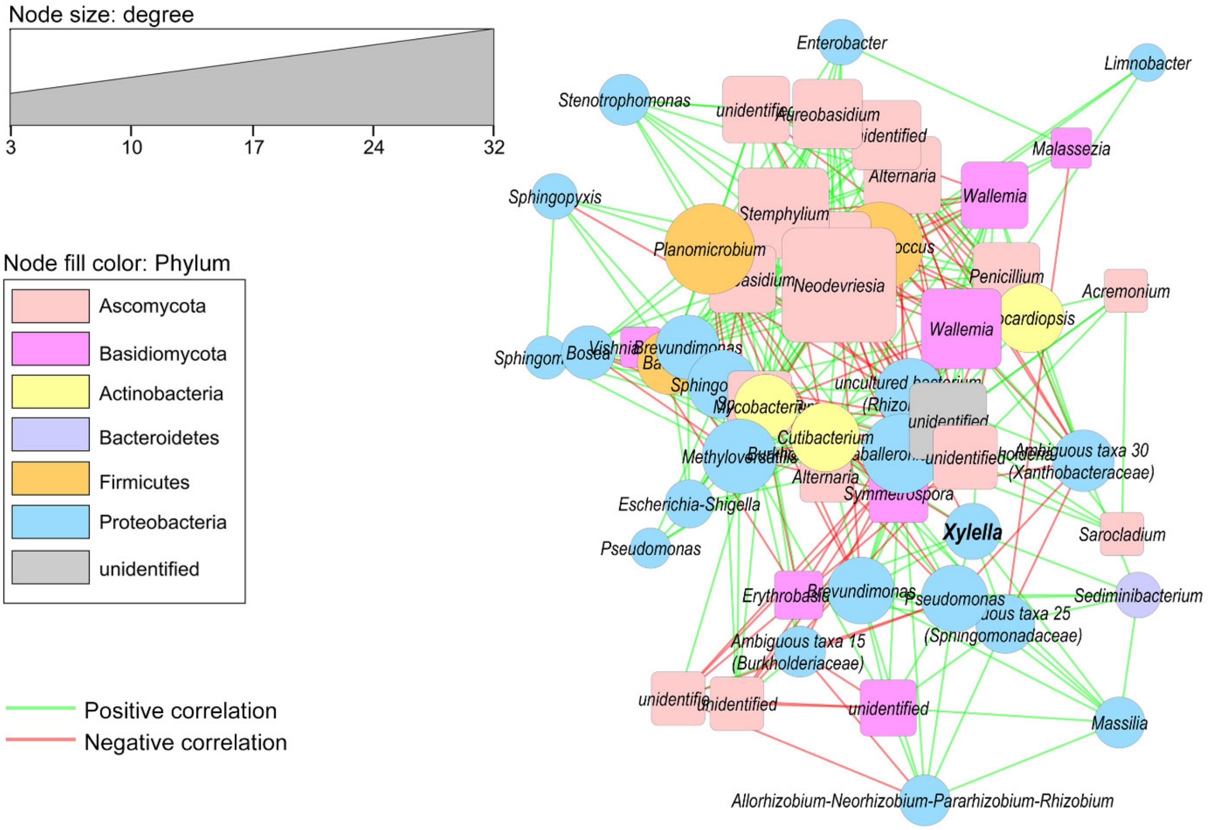

Fig. S6 Original co-occurrence network. The network was arranged by the "edge-forced spring embedded" layout, additionally weighted by the *p*-value.

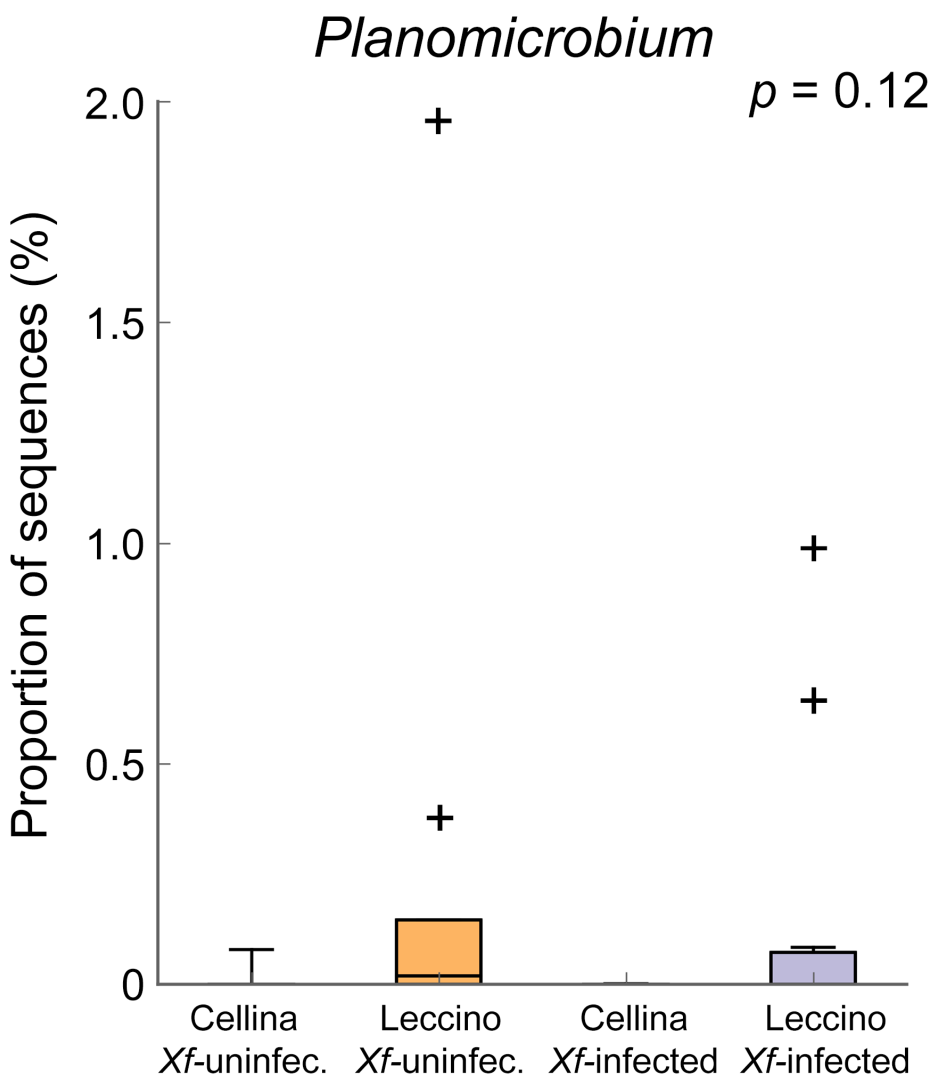

Fig. S7 Distribution of the OTU identified as *Planomicrobium* among sample groups. This OTU was identified as a "hub species" in the microbial network of the infected "Leccino" leaf endophytes (Fig. 6, Fig. S6). P-value according to Kruskal-Wallis test (FDR-corrected). *Xf*= *Xylella fastidiosa*; "+" signs indicate outliers.
